# Reactivation of the silenced *BASP1* gene suppresses oncogenic WNT signaling in human colorectal cancer cells

**DOI:** 10.1073/pnas.2524159123

**Published:** 2026-03-05

**Authors:** Leonie I. Weber, Lea E. Timpen, Anna-Sophia Egger-Hörschinger, Philemon Schöpf, Nesin D. Ayhan, David Demmel, Madlen Hotze, Yang Zhang, Mahdi Mehrabi, Kane Puglisi, Eduard Stefan, Nassim Ghaffari-Tabrizi-Wizsy, José M. Ramos-Pittol, Marcel Kwiatkowski, Markus Hartl

**Affiliations:** ^a^Institute of Biochemistry, Faculty of Chemistry and Pharmacy, University of Innsbruck, Innsbruck 6020, Austria; ^b^Center for Molecular Biosciences, University of Innsbruck, Innsbruck 6020, Austria; ^c^Otto Loewi Research Center, Division of Immunology, Medical University of Graz, Graz 8010, Austria; ^d^Institute of Molecular Biology, Faculty of Biology, University of Innsbruck, Innsbruck 6020, Austria; ^e^Tyrolean Cancer Research Institute, ONCOkinase laboratory, Innsbruck 6020, Austria

**Keywords:** gene regulation, CRISPR, CRISPR-Cas, v-Myc avian myelocytomatosis viral oncogene homolog (MYC), Traf2 and Nck-interacting kinase (TNIK), tumor formation

## Abstract

Starting from human colon cancer cells showing aberrant WNT/β-catenin/TCF signaling, hyperactivated *MYC,* and silenced *BASP1*, we generated stable cell lines overexpressing *BASP1*, either ectopically, or by reactivating the dormant *BASP1* promoter using a lentiviral CRISPR-based system. *BASP1* encodes a neuronal signaling protein and transcriptional corepressor, from which tumor-suppressive functions have been described in avian cell systems and in multiple human cancer cell types. Proteome and transcriptome analyses revealed activation of several tumor and metastasis suppressors in BASP1-expressing cells, which also show strong repression of the transformed phenotype in terms of contact inhibition, anchorage-independent growth, and tumor formation. Cells with reactivated BASP1 display a flat and differentiated morphology with enhanced migratory potential, accompanied by expression of multiple genes implicated in actin polymerization, focal adhesion, and neuronal migration. Furthermore, MYC protein expression is substantially repressed due to BASP1-mediated transcriptional *MYC* downregulation involving BASP1 interaction with β-catenin and binding to the *MYC* promoter. Upon *BASP1* activation, multiple key proteins of the canonical WNT signaling pathway become suppressed. One of these BASP1 targets is the protein kinase TNIK catalyzing phosphorylation of TCF7L2, the latter required for transcriptional *MYC* activation. Results obtained with a preclinical TNIK inhibitor in human colorectal cancer cells show efficient abrogation of MYC expression and consequently impaired dimerization with its interaction partner MAX. The antagonistic BASP1 effect on MYC and the MYC dependency on TNIK could enhance the development of strategies to interfere with oncogenic functions of the cancer driver MYC.

## Significance

Due to its pleiotropic functions in gene regulation, chromatin remodeling, and metabolism, MYC family members have a fundamental impact on cell growth control and proliferation. Aberrant activation of MYC provokes derailed cell signaling and malignant cell transformation in most human tumors. Therefore, genetic or pharmacological MYC inhibition represents a rational therapeutic approach. We have achieved this in colorectal cancer cells by genetically inducing transcriptional activation of *BASP1* whose protein product interferes with MYC activity. This leads to suppression of tumor formation accompanied by WNT signaling repression and transcriptional downregulation of the effector target *MYC*. Furthermore, blocking WNT-associated protein kinase TNIK using a small molecule also results in transcriptional *MYC* repression, thereby expanding the tool repertoire to inhibit this oncogenic transcription factor.

MYC is a gene regulator with oncogenic potential and represents the hub of a network controlling the expression of numerous genes. Thereby, MYC regulates cellular processes like growth, proliferation, differentiation, metabolism, pluripotency, and apoptosis (1, 2). Overexpression of MYC causes deregulation of cell cycle progression, metabolism, differentiation, and angiogenesis, which contributes to neoplastic transformation. In most human cancers like leukemia, lymphoma, or carcinoma, the *MYC* gene is aberrantly activated by transcriptional deregulation, gene amplification, chromosomal translocation, or posttranslational modification (1, 3). Amplification of the *MYC* gene also occurs in colorectal cancer, besides transcriptional *MYC* activation caused by the wingless/int (WNT)/β-catenin signaling pathway (4–6). Thereby, the nuclear effector TCF7L2, formerly known as TCF-4 (7), represents an oncogenic transcriptional regulator of the *MYC* and cyclin D1 genes (5, 8, 9).

We have previously reported that transcription of the *BASP1* gene is strongly and specifically repressed in avian cells transformed by the v-*myc* oncogene. Moreover, we showed that the presence of ectopic BASP1 leads to inhibition of v-*myc*-induced cell transformation (10, 11). The *BASP1* gene encodes the brain acid-soluble protein 1, which is particularly abundant in nerve terminals during brain development and implicated in neurite outgrowth, maturation of the actin cytoskeleton, and organization of the plasma membrane, but is also expressed in various other tissues (12). Upon protein kinase C-mediated phosphorylation, BASP1 is translocated into the nucleus via a bipartite nuclear localization signal, where this protein acts as transcriptional corepressor (13–15). Blocking BASP1-mediated transcriptional repression is essential during reprogramming of differentiated cells into induced pluripotent stem cells (16).

*BASP1* is downregulated in multiple mammalian tumors like carcinoma, acute and chronic lymphocytic leukemia, or melanoma, which is achieved by direct transcriptional repression, microRNA-guided downregulation, or promoter methylation (17–21). Accordingly, most human tumors with downregulated *BASP1* display aberrantly elevated *MYC* expression (12). Tumor-suppressive functions of BASP1 were observed in multiple human cancer types such as acute myeloid leukemia (20), thyroid cancer (22), breast cancer (23), gastric cancer (24), pancreatic cancer (25), prostate cancer (26), neuroblastoma (27), or glioma (28, 29). Recently, we have shown that lipid-based formulations containing a BASP1 peptide have antiproliferative effects on distinct human leukemia and gastrointestinal cell lines (30). On the other hand, BASP1 levels are paradoxically high in cervical cancer cells where the *BASP1* locus on chromosome 5 is amplified (31). Furthermore, it has been reported that BASP1 could even promote tumor growth in these cancer cells (32). Several cervical cancer cell lines contain high *BASP1* but low *MYC* levels, suggesting that the growth-inhibiting function of BASP1 is restricted to tumor cells with aberrantly elevated *MYC* expression levels, as it is the case for most leukemia cells (33). BASP1 is also upregulated and associated with poor survival in head and neck squamous cell carcinoma patients (34) and in reported cases of lung adenocarcinoma (35, 36). These findings implicate that BASP1 could have a dichotomic role in tumor suppression and oncogenesis depending on the cancer cell type.

Based on these findings, we investigated here the consequences of overexpressed BASP1 in human colorectal cancer cells, which are featured by high MYC expression and a transcriptionally suppressed *BASP1* gene. BASP1 was either ectopically expressed or the endogenous silenced *BASP1* gene reactivated using a CRISPR-based artificial transcriptional activator. We show that in this human cancer cell system, BASP1 severely interferes with MYC activity and oncogenicity, as it has been previously observed in avian fibroblasts (10, 11). BASP1-expressing colon cancer cells display a pronounced nononcogenic phenotype with features typical for differentiated cells but also enhanced migration compared to the parental cell line. BASP1-mediated MYC inhibition is due to severely impaired WNT signaling representing the major tumorigenic pathway in this human cancer type.

## Results

### Identification of Clinically Relevant Cancer Cell Lines Using Bioinformatics.

To identify patient-relevant human tumor cell lines featured by high *MYC* and low *BASP1* expression levels, expression data from human cancer cell lines and from patient cohorts were retrieved from relevant databases and compared to each other. For this reason, the computational tool *rna*Ratio was developed (*SI Appendix*, Fig. S1*A*). Expression levels and expression ratios of cell lines and patient data were visualized by box- and scatter blots (*SI Appendix*, Figs. S1 *B* and *C*). Multiple cell lines fulfilling the criteria of high *MYC* and low *BASP1* expression were identified including the erythroleukemia cell line K562, in which ectopic BASP1 expression leads to a differentiated phenotype (14). In addition, bowel tumors of patients and the herewith associated colorectal cancer cell line SW480 exhibit high *MYC*/*BASP1* expression ratios (*SI Appendix*, Fig. S1*C*). SW480 is originally derived from a colorectal adenocarcinoma, representing a suitable model system to investigate the molecular and functional diversity of colon cancer cells. SW480 originate from epithelial cells and consist of three distinct subpopulations, namely resident cancer stem cells, migratory stem cells, and high-relapse cells, which are also found in patient-derived cancers in distinct spatial distributions relative to the tumor microenvironment (37). Due to the heterogeneity and plasticity of SW480, the underlying molecular signatures are reminiscent of clinical colon cancer specimens, and make SW480 suitable for studying as a representative of patient-derived carcinoma in vitro. Furthermore, SW480 are featured by constitutively activated WNT signaling and gene expression profiles mirroring their stem-like properties (37), and these cells contain an intact but silenced *BASP1* gene. For these reasons, SW480 were found to represent an appropriate model system for challenging the elevated expressed MYC oncoprotein with ectopic or reactivated *BASP1* expression.

### Ectopic BASP1 Leads to Lower MYC Expression and Impaired Transformed Phenotype.

**Fig. 1.**
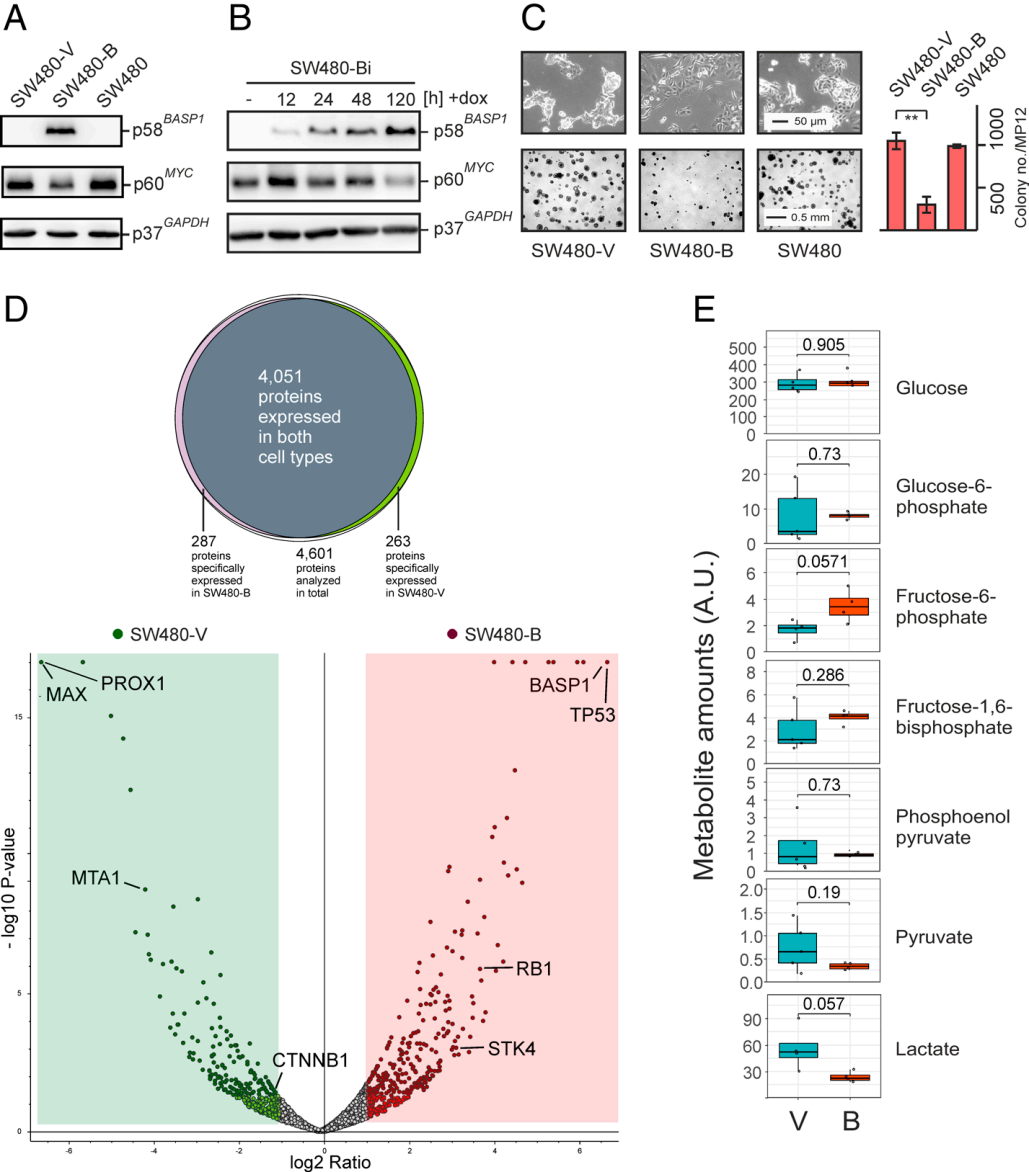
MYC attenuation and differential protein expression in SW480 cells upon ectopic BASP1 expression. SW480 cells were transfected with the empty pcDNA3 expression vector or with pcDNA3-BASP1, and isogenic cell lines (SW480-V, SW480-B) were obtained after geneticin (G418) selection. (*A*) Immunoblot analysis showing expression of ectopic BASP1, endogenous MYC, and GAPDH. (*B*) Immunoblot of SW480 cells stably transfected with the construct pAK-Tol2-TRE-BASP1 allowing inducible BASP1 expression (SW480-Bi). Cell extracts were prepared after the indicated time points upon doxycycline (dox) addition. (*C*) Ectopic human BASP1 causes morphological changes (micrographs of cells at the *Top*) and a reduced transformed phenotype (colony formation in soft agar at the *Bottom*). Vertical bars show SD from independent experiments. Statistical significance was assessed by using an unpaired Student *t* test (**P < 0.01). (*D*) Proteome analysis by liquid chromatography-mass spectrometry (LC–MS) measurement of SW480-B and SW480-V cells (n = 5 independent experiments). *Top*: Venn diagram of identified proteins in SW480-B and SW480-V cells. *Bottom*: Volcano plot of proteins specifically overexpressed in SW480-V (light green) or SW480-B cells (pink). Proteins highlighted in green and red have a fold change of at least 2.0 and a *P*-value < 0.05. (*E*) Analysis of simultaneously extracted glycolytic metabolites of SW480-V and SW480-B cells (V/B). Significances determined by the Wilcoxon rank-sum test are shown in the boxes. Values of arbitrary units (A.U.) are shown at the *Left*.

To test how SW480 cells respond to ectopically expressed BASP1, SW480 were transfected with the plasmid pcDNA3-BASP1 containing the coding region of the human *BASP1* gene. Several cell lines stably expressing BASP1 were established upon geneticin selection and one of them termed SW480-B was used for further analyses. As control, a cell line from empty pcDNA3 vector transfection was established (SW480-V). Immunoblot analysis confirmed efficient expression of ectopic BASP1 in SW480-B, whereas in SW480 or SW480-V, the BASP1 protein was not detected (Fig. 1*A*). Furthermore, reduced MYC protein levels were seen in SW480-B cells. To test if MYC downregulation is a direct effect of ectopic BASP1, the cell line SW480-Bi was generated allowing inducible *BASP1* expression from the construct pAK-Tol2-TRE-BASP1. After doxycycline addition, *BASP1* was activated leading to a decline of MYC protein expression during five days (Fig. 1*B*) thus corroborating a correlation between ectopic BASP1 expression and MYC downregulation.

BASP1-expressing SW480 display a less refractile and more adherent morphology also giving rise to a significantly lower number of colonies in soft agar, which indicates a lower capacity of anchorage-independent growth (Fig. 1*C*). Likewise, ectopic BASP1 expression in the isogenic cell line SW620, derived from a metastatic tumor, led to reduced proliferation and agar colony formation (*SI Appendix*, Fig. S2). To analyze SW480-B at a molecular level, proteome and metabolome analyses were carried out using liquid chromatography coupled to mass spectrometry (LC–MS) in comparison with the control cell line SW480-V (Fig. 1*D*). From 4,601 identified proteins, 287 and 263 were exclusively present in SW480-B or SW480-V, respectively. 509 proteins were found to be more abundant in SW480-B including the tumor suppressors p53 (TP53) and retinoblastoma-associated protein 1 (RB1). Among the 370 proteins less abundant in SW480-B are the MYC dimerization partner MAX, and the metastasis-associated protein 1 (MTA1), the latter representing an oncogenic target of MYC (38). Surprisingly, the MYC protein could not be detected in this analysis possibly due to its high proportion of intrinsically disordered regions (39), which may have resulted in inefficient fragmentation thereby reducing MS detection rates.

Besides, differential expression of several WNT signaling proteins was observed in SW480-B cells such as moderately downregulated β-catenin (CTNNB1) or upregulated STK4, the latter representing a protein kinase destabilizing CTNNB1 (40). Likewise, the transcription factor PROX1 representing a nuclear effector in WNT signaling (41) is repressed in SW480-B cells. Pathway analysis of the underlying genes using gene ratios and gene clusters revealed differential expression of focal adhesion and cell substrate junction proteins (*SI Appendix*, Fig. S3). The corresponding proteins are required for cadherin and actin filament binding, which is in accordance with a central BASP1 function in promoting nerve cell sprouting (42). Thereby, the actin cytoskeleton needs to be modified, a process which could also account for the observed morphological changes in the SW480-B cell line (Fig. 1*C*). Furthermore, we examined if the reduced transformed phenotype may have an impact on lactate production, which is typically enhanced in tumor cells. For this reason, simultaneously extracted metabolites (43) were measured by mass spectrometry. Analysis of multiple glycolytic metabolites revealed reduced lactate concentration in SW480-B cells (Fig. 1*E*) thereby correlating with lower MYC expression (Fig. 1*A*). Otherwise, MYC drives aerobic glycolysis also known as the Warburg effect (44).

To test the observed proliferation-inhibitory effect of BASP1 in an independent cell system, the *BASP1* gene in the human breast cancer cell line MCF7 was inactivated using standard CRISPR technology (*SI Appendix*, Fig. S4). Compared to SW480 cells, MCF7 are featured by significant higher *BASP1* expression (*SI Appendix*, Fig. S1*C*). Genetic modification of *BASP1* resulting in the cell line MCF7/BASP1-ko led to disruption of the *BASP1* translation start site, thereby preventing BASP1 protein synthesis (*SI Appendix*, Figs. S4 *A* and *B*), which was verified by immunoblot analysis (*SI Appendix*, Fig. S4*C*). MCF7/BASP1-ko cells lacking BASP1 display a higher proliferation rate (*SI Appendix*, Fig. S4*D*) and enhanced agar colony formation (*SI Appendix*, Fig. S4*E*) thus corroborating the antiproliferative function of BASP1.

### Reactivating Silenced BASP1 Leads to Transcriptional MYC Downregulation.

**Fig. 2.**
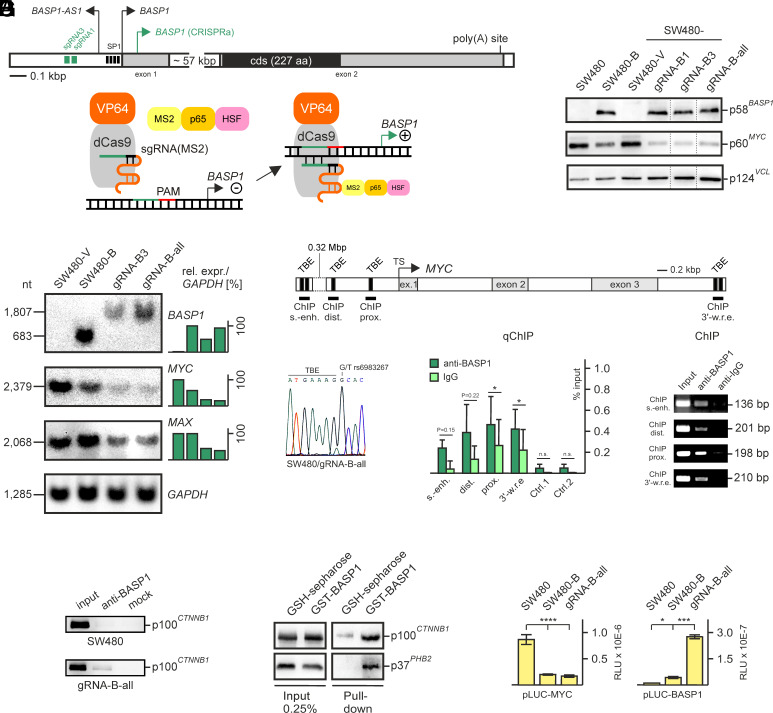
Reactivation of the silenced *BASP1* gene in the human cancer cell line SW480. (*A*) Structure of the silenced human *BASP1* gene and strategy how *BASP1* transcription is induced by CRISPRa using a synergistic activation mediator (SAM), which consists of VP64 and MS2-p65-HSF (47). The two *BASP1* exons are shaded in gray, the protein coding sequence in black. Transcription start sites for the regular *BASP1* mRNA and the long noncoding antisense *BASP1* RNA (*BASP1-AS1*) are indicated by black arrows. The positions of two sgRNA binding sites are depicted as green bars, and the mapped transcription start of the CRISPRa-induced mRNA by a green arrow. The applied artificial transcription factor is based on the CRISPR/Cas9 principle using a mutant Cas9 (dCas9) protein fused to an artificial transcriptional activator (VP64). (*B*) Immunoblot analysis to test for protein expression using extracts from SW480 and BASP1-expressing SW480 cell lines as indicated, and antibodies directed against BASP1, MYC, or vinculin (VCL). The dotted lines mark splicing sites in the blots, from which two redundant lanes have been removed. (*C*) Northern analysis to monitor *BASP1*, *MYC*, and *MAX* mRNA expression in the cell lines SW480-gRNA-B3 and SW480-gRNA-B-all in comparison with SW480-B and SW480. The mRNA nucleotide (nt) sizes of endogenous *BASP1*, ectopic *BASP1*, *MYC*, *MAX*, and *GAPDH* are given on the *Left*. Expression levels (%) were determined using the program ImageQuant TL and are depicted as green bars in relation to *GAPDH*. (*D*) Binding of BASP1 to *MYC* regulatory regions determined by chromatin immunoprecipitation analysis (ChIP). Locations of the tested regions are depicted on the *Top*, which contain TCF binding sites (TBE), namely the superenhancer (48), the 5’-promoter (distal and proximal) (5), and the 3’-region (49). The cancer-associated SNP in the superenhancer is retained in the applied SW480-gRNA-B-all cells (*Left* panel). Quantitative (q) and qualitative ChIP using a human BASP1-specific peptide antibody (anti-BASP1) or an IgG control, and fixed and sheared chromatin from SW480-gRNA-B-all cells. Two nonregulatory human DNA segments were used a negative control (Ctrl.) as described (50). Vertical bars show SE of the mean (SEM) from independent experiments (n ≥ 3). Statistical significance was assessed by using an unpaired Student *t* test (*P < 0.05). (*E*) Coimmunoprecipitation (Co-IP) analysis using protein extracts from SW480 and SW480-gRNA-B-all cells and anti-BASP1 as first antibody to test for physical interaction between BASP1 and β-catenin (CTNNB1). (*F*) Pull-down of CTNNB1 after interaction with a recombinant GST-BASP1 fusion protein. Lysates from SW480-gRNA-B-all cells prepared under native conditions were loaded onto GST-BASP1-charged glutathione (GSH)-sepharose or onto empty GSH-sepharose beads. Bound cellular proteins were eluted under denaturing conditions, subjected to SDS-PAGE (10% w/v), and immunoblotted using antibodies directed against human CTNNB1 or the prohibitin (PHB) paralog PHB2, the latter serving as a control (51). As input control, 0.25 % of the lysates were used. The band in the GSH-sepharose pulldown migrating above 100 kDa results from an unspecific protein. (*G*) Luciferase reporter activities in relative light units (RLU) showing suppression of *MYC* promoter activity in the presence of BASP1 (*Left* panel), and *BASP1* promoter activation in SW480-gRNA-B-all cells expressing the dCas9-VP64 transactivator and *BASP1*-specific sgRNAs (*Right* panel). Vertical bars show SD from independent experiments. Statistical significance was assessed by using an unpaired *t* test (*P < 0.05, ***P < 0.001, ****P < 0.0001).

To reexpress the endogenous *BASP1* gene in SW480 cells, a CRISPR-based strategy was pursued using three lentiviral vectors encoding I) a nuclease-deficient Cas9 enzyme (dCas9) fused to the strong transcriptional activator VP64 (dCas9-VP64) (45), II) specific guide RNAs to direct dCas9-VP64 to the *BASP1* promoter, and III) the MS2-P65-HSF1 (SAM) complex for synergistic transcriptional activation (46) (Fig. 2*A*). The vectors were first transfected into HEK293T cells for virus production, and after three viral infection rounds, the stable cell lines SW480-gRNA-B1, SW480-gRNA-B3, and SW480-gRNA-B-all could be established. All of them efficiently express the BASP1 protein at levels comparable to ectopic BASP1 from SW480-B, whereas in SW480-V or SW480, no BASP1 was detectable, as expected. Likewise, the MYC protein was downregulated in the genetically modified cells, even to a stronger extent compared to SW480-B (Fig. 2*B*). To verify if BASP1 protein expression results from CRISPR-mediated transcriptional *BASP1* activation, Northern analysis was performed using total RNAs isolated from SW480-gRNA-B3 and SW480-gRNA-B-all cells and compared with those from SW480-V and SW480-B cells (Fig. 2*C*). A 1.8-kb *BASP1*-specific mRNA was efficiently activated in the first two cell lines at levels comparable to the 0.7-kb ectopic *BASP1* transcript expressed from the pcDNA3-BASP1 vector in SW480-B. To map the start site of CRISPR-activated *BASP1* mRNA transcription, 5’-rapid amplification of cDNA ends (5’RACE) was performed showing that transcription starts within exon 1 but 137 nucleotides downstream of the regular transcription start site (Fig. 2*A*). Reciprocally to *BASP1* activation, significant mRNA downregulation was observed for *MYC*, and for *MAX* encoding the MYC-dimerization partner (Fig. 2*C*).

The WNT signaling pathway activates the *MYC* promoter in colon cancer cells, which is mediated by the transcription factor complex β-catenin/TCF7L2 (5, 7, 52). Due to transcriptional *MYC* downregulation in BASP1-expressing SW480 cells, the presence of BASP1 on characteristic *MYC* promoter segments containing functional TCF binding sites (TBE) was tested. Besides the originally described distal and proximal TBE elements in the 5’-non-transcribed MYC region (5), additional TBE clusters were tested. One of them is located 1.4 kbp downstream of the *MYC* transcriptional stop site (49), and another present in the super-enhancer region localized 0.32 Mbp upstream of *MYC* (48) (Fig. 2*D*). The latter region contains a high cancer risk-associated single-nucleotide polymorphism (SNP) juxtaposed to a TBE site (G > T) (48, 53), which is also present in SW480 and in the BASP1-expressing derivative SW480-gRNA-B-all, as determined by DNA sequencing (Fig. 2*D*). Quantitative and qualitative chromatin immunoprecipitation (ChIP) analyses using formaldehyde-fixed chromatin prepared from SW480-gRNA-B-all cells and a BASP1-specific antibody revealed significant BASP1 binding on DNA fragments containing the proximal (prox.) and the downstream (3’-w.r.e.) TCF7L2 binding sites (Fig. 2*D*). Weaker binding was detected on fragments encompassing the distal (dist.) and the super-enhancer (s.-enh.) TBE elements, whereas no significant binding was observed on two genomic nonpromoter regions, which were used as control. This interaction with the *MYC* gene suggests that BASP1 may have a direct impact on *MYC* expression.

The BASP1 effect on transcriptional control is corroborated by specific protein–protein interaction between BASP1 and β-catenin (CTNNB1), which was detected by coimmunoprecipitation (CoIP) analysis using lysates from SW480-gRNA-B-all cells (Fig. 2*E*), and by protein pulldown using a recombinant GST-BASP1 fusion protein (Fig. 2*F*). These results suggest that BASP1 physically interacts with CTNNB1 and may bind the *MYC* promoter in context of a possible BASP1/CTNNB1/TCF7L2 complex. Moreover, luciferase reporter assays using a *MYC* promoter construct (pLUC-MYC) encompassing the distal and proximal TBE sites showed strong downregulation in the presence of BASP1 (Fig. 2*G*) and demonstrate the effect of BASP1 on transcriptional *MYC* repression. Conversely, a *BASP1* promoter plasmid (pLUC-BASP1) was activated in the presence of ectopic BASP1 (SW480-B) due to reduced levels of MYC, which otherwise suppresses *BASP1* transcription (10). In case of SW480-gRNA-B-all cells, a strong synergistic effect in transcriptional activation was observed due to the presence of the *BASP1* promoter-guided dCas9-VP64 activator, thereby finally confirming the functionality of the applied CRISPR-activation system.

### Biological Properties of BASP1-Expressing Colon Cancer Cells.

**Fig. 3.**
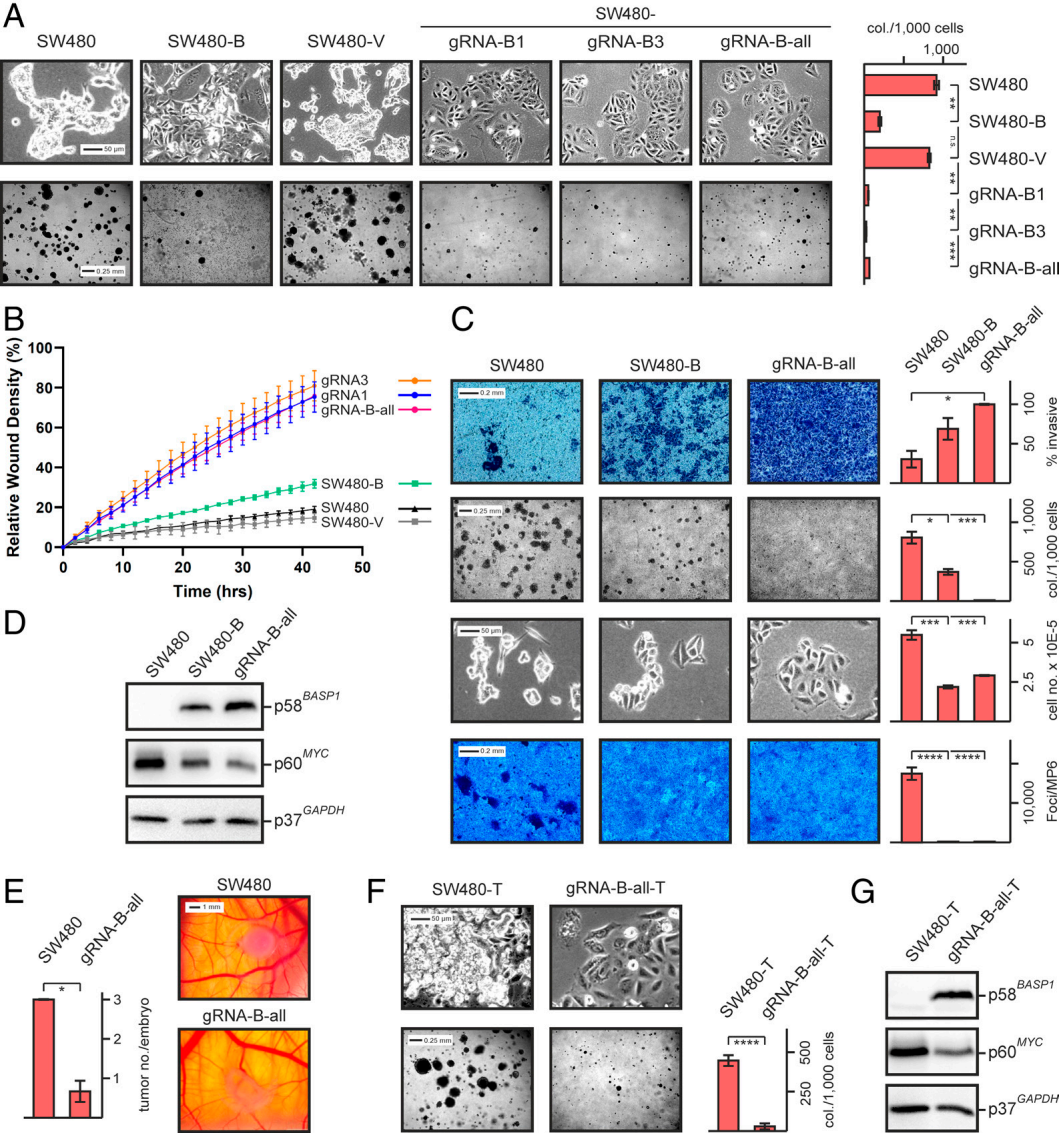
Biological effects of BASP1-expressing SW480. (*A*) Upper panel: micrographs of SW480, SW480-B, SW480-V, and SW480 cells with reactivated endogenous *BASP1* (gRNA-B1, -B3, -B-all). Lower panel: colony formation after 10 d in soft agar upon seeding each 7,500 cells into MP12 wells. Vertical bars below show SD from independent experiments. Statistical significance was assessed by using an unpaired Student *t* test (**P < 0.01, ***P < 0.001, n.s., not significant). (*B*) Cell migration monitored after scratching 80%-confluent cells using an IncuCyte system. (*C*) Direct comparison of invasion, clonogenicity, proliferation, and contact inhibition. *First* panel: cell invasion assay of SW480 and BASP1-expressing SW480. Cells passing the extracellular matrix were stained and quantified using the program ImageQuant TL. *Second* panel: invaded cells were subsequently tested for their clonogenicity using an agar colony assay. *Third* panel: proliferation measurement where each 50,000 cells were seeded into MP12 wells and incubated for 3.5 d. Then, cells were dissociated with trypsin-EDTA and their total numbers determined. Fourth panel: test for focus formation. After having reached confluency, cells were overlaid with agar and incubated for 10 d. Vertical bars show SD from counted triplicates. Statistical significance was assessed by using an unpaired *t* test (*P < 0.05, **P < 0.01, ***P < 0.001, ****P < 0.0001). (*D*) Immunoblot analysis to verify BASP1 and MYC expression. (*E*) Tumor formation monitored by an ex ovo chorioallantoic membrane (CAM) assay. SW480 or BASP1-expressing SW480 were inoculated in triplicate onto the CAMs of 9-d chicken embryos and allowed to develop tumors for 4 d. *Left* panel: quantification of developed tumors in both cell types. *Right* panel: Morphologies of tumors derived from the tested cell types. (*F*) Micrographs of cells isolated from the relevant tumors (*Upper* panel) and respective colony formation potentials (*Lower* panel). (*G*) Immunoblot analysis to monitor protein expression in cells derived from individual tumors (SW480-T, SW480-gRNA-B-all-T).

Like SW480-B cells, SW480-gRNA-B cells are featured by a flat uniform cell morphology, even more pronounced and clearly different from the parental SW480 cell line. Furthermore, SW480-gRNA-B have completely lost their capacity of anchorage-independent growth (Fig. 3*A*). Despite these properties, BASP1-expressing SW480 display higher migratory capacity compared to SW480 or SW480-V as determined by a scratch/wound healing assay (Fig. 3*B*). To test if this higher cell migration potential correlates with increased invasion capacity, SW480, SW480-B, and SW480-gRNA-B-all were seeded onto an artificial extracellular matrix (ECM) and invaded cells were quantified after three days. This analysis shows that BASP1 expression increased the known invasive potential of SW480 (37) (Fig. 3*C*). Nevertheless, these invading cells did not grow anchorage-independently, demonstrating that invasion and clonogenicity are distinguishable properties. Furthermore, BASP1-expressing cells had lower proliferation rates and contact-inhibited monolayer growth, whereas parental SW480 cells were featured by a loss of contact inhibition resulting in focus formation under agar overlay (Fig. 3*C*). Protein analysis confirmed sustained MYC downregulation in BASP1-expressing cells (Fig. 3*D*).

To test if reactivated BASP1 also interferes with the reported tumorigenic potential of SW480 cells (54), SW480 and SW480-gRNA-B-all were tested in vivo using an ex ovo chicken chorioallantoic membrane (CAM) assay. This is a procedure to monitor cell migration and invasion through basement membranes for mimicking tumor invasion and metastasis (55). SW480 cells seeded onto the CAM led to efficient formation of compact tumors, whereas BASP1-expressing SW480 only induced few tumors with diffuse morphology (Fig. 3*E*). Histochemical comparison of sliced tumor tissue derived from both cell types revealed more cell nucleic structures in SW480-derived tumors manifested by stronger hematoxylin staining but on the other hand, the rare tumors derived from SW480-gRNA-B-all cells displayed a higher degree of invasiveness and angiogenesis (*SI Appendix*, Fig. S5*A*). On the other hand, immunohistochemical analysis revealed that in SW480-derived tumors the Ki-67 (MKI67) protein, representing a mitosis indicator and tumor aggressiveness marker, is significantly stronger expressed (*SI Appendix*, Fig. S5*B*). Accordingly, cells dissociated from individual tumors caused by SW480 or SW480-gRNA-B-all differed clearly in terms of morphology, density, colony formation in soft agar (Fig. 3*F*), and MYC expression (Fig. 3*G*) confirming that nonclonogenicity and MYC downregulation were retained in the analyzed tumor cells. In summary, *BASP1* reactivation in colon cancer cells abrogates the transformed phenotype in terms of contact-inhibition, colony/focus formation, and tumor formation, although these apparently nontransformed cells display enhanced cell migration and invasion.

### BASP1 Expression Affects Typical Signaling Pathways in Colon Cancer Cells.

**Fig. 4.**
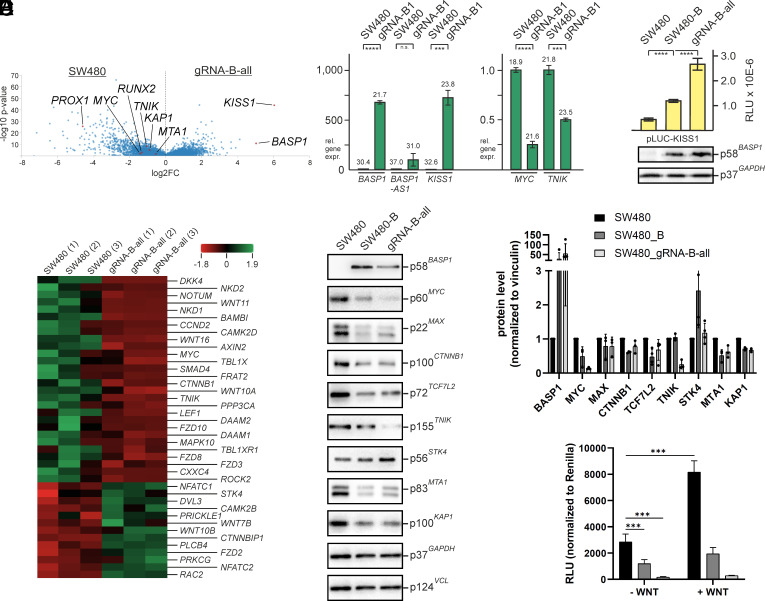
Gene profiling of BASP1-expressing colon cancer cells. (*A*) Volcano blot visualizing differentially expressed mRNAs of SW480 and SW480-gRNA-B-all cells determined by RNA-Seq analysis. (*B*) Quantitative PCR (qPCR) to verify differential expression of the selected genes *BASP1*, *MYC*, *KISS1*, or *TNIK* (***P < 0.001, ****P < 0.0001, n.s. not significant). (*C*) Luciferase reporter assay showing *KISS1* promoter activity in the presence of BASP1. (*D*) Heat map of genes relevant for the WNT signaling pathway showing differential expression in triplicate samples of SW480 versus SW480-gRNA-B-all cells. (*E*) Representative immunoblot analysis to monitor expression of key proteins from the WNT signaling pathway (*Left* site), and quantification of triplicate blots using the program Image-Quant TL (*Right* site). (*F*) Inhibition of WNT signaling by BASP1. To test for WNT signaling activity, a TOP/FOP-Flash assay was conducted using SW480, SW480-B, and SW480-gRNA-B-all cells with (+) or without (−) addition of recombinant WNT3a ligand for pathway stimulation. The ratios TOP (reporter with TBE)/FOP (reporter with mutated TBE) normalized to Renilla luciferase are depicted on the y-axis (***P < 0.001).

To compare SW480, SW480-B, and SW480-gRNA-B-all cells on a molecular level, their transcriptomes were analyzed by RNA sequencing (RNA-Seq) using in triplicate-isolated total RNAs (Fig. 4). Comparison of 25,671 genes in the SW480 and SW480-gRNA-B-all transcriptomes revealed that 1,131 or 803 mRNAs are specifically activated by a factor >2 in SW480 or SW480-gRNA-B-all, respectively. Similar ratios were observed in a comparison between SW480 and SW480-B cells (25,759 genes, 659 or 726 > 2-fold-activated, respectively).

Among the strongest activated genes in BASP1-expressing cells is the metastasis suppressor *KISS1* (56–58), whereas multiple genes associated with WNT signaling are downregulated including *PROX1*, *RUNX2*, *MYC*, or Traf2 and Nck-interacting kinase (*TNIK*) (Fig. 4*A*). As mentioned above, PROX1 is a nuclear effector in WNT signaling (41), and RUNX2 represents an epigenetic regulator increasing the metastatic properties of SW480 cells by promoting epithelial to mesenchymal transition (EMT) as a consequence of enhanced WNT signaling (37, 59). TNIK is responsible for phosphorylation and activation of TCF7L2 (60), a major nuclear WNT effector and transcriptional *MYC* activator (5). Likewise, genes promoting tumorigenesis and metastasis progression are downregulated in BASP1-expressing cells like *MTA1* or *TRIM28*. MTA1, also identified in our proteome analysis (Fig. 1*D*) is a component of the nucleosome remodeling deacetylase complex NuRD, and TRIM28 represents the KRAB-associated protein 1 (KAP1) (61).

Differential expression of selected genes was confirmed by qPCR using cDNA transcribed from total RNA (Fig. 4*B*). While *BASP1* and *KISS1* are strongly expressed in SW480-gRNA-B-all cells, *MYC* and *TNIK* mRNAs were downregulated as expected. The previously described *BASP1* antisense RNA (*BASP1-AS1*) representing a long noncoding RNA (Fig. 2*A*) positively regulating stemness and pluripotency (62) was not significantly activated by *BASP1* promoter-guided dCas9-VP64 (Fig. 4*B*). Besides transcriptional repression, BASP1 may also stimulate gene transcription as suggested by observed equal proportions of differentially regulated genes. As proof of principle, a 3.1-kbp fragment of the human *KISS1* promoter (63, 64) was tested for transactivation in SW480 and BASP1-expressing SW480 using an appropriate reporter construct (pLUC-KISS1). The presence of BASP1 led to strong transcriptional activation (Fig. 4*C*), demonstrating that BASP1 can also confer positive gene regulation, possibly upon interaction with SP1 or other transcription factors as reported previously for the *KISS1* gene (65–67).

Transcriptome analyses revealed that multiple genes associated with WNT signaling are downregulated in BASP1-expressing SW480 (Fig. 4*D*), which was verified on the protein level for MYC, MAX, CTNNB1, TCF7L2, and TNIK in addition to the tumorigenesis drivers MTA1 and TRIM28 (KAP1) (Fig. 4*E*). On the other hand, upregulation of CTNNB1-destabilizing STK4, as already seen in the proteome analysis (Fig. 1*D*), was confirmed. Gene set enrichment analysis (GSEA) also showed downregulation of multiple genes associated with WNT signaling in SW480-gRNA-B-all (*SI Appendix*, Fig. S6*A*). To proof direct interference of BASP1 with this oncogenic pathway, a WNT luciferase reporter system was applied to test for transcriptional activation of a TCF-responsive promoter in SW480, SW480-B, and SW480-gRNA-B-all cells (Fig. 4*F*). In presence of BASP1, WNT signaling was strongly impaired. While addition of the WNT3a factor led to significant increase of WNT pathway activity in SW480 cells, BASP1-expressing cells showed no increased WNT activity upon WNT3a stimulation. Further data analysis of the transcriptomes confirmed a strong preponderance of genes specifically involved in positive regulation of actin polymerization, cell migration, and adhesion in BASP1-expressing cells (*SI Appendix*, Fig. S6) in accordance with the above monitored cell biological functions (Fig. 3). In addition, genes implicated in neurogenesis and neuronal differentiation are activated upon BASP1 expression reflecting the original neuronal function of BASP1 (*SI Appendix*, Fig. S6*B*).

In colon cancer, high WNT signaling frequently correlates with enhanced activity of the mitogen-activated protein kinase (MAPK) pathway (68) including its nuclear effector AP-1 (activating protein-1), a dimeric transcription factor complex consisting of JUN and FOS proteins (69). The transcriptome analysis showed potential activation of MAPK signaling in BASP1-expressing cells (*SI Appendix*, Fig. S6*A*). Therefore, relevant mRNA and protein expression studies were performed in combination with protein–protein interaction studies and reporter gene assays. These analyses revealed strong *FOS* activation in BASP1-expressing cells (*SI Appendix*, Figs. S7 *A*–*C*), and a physical interaction between the BASP1 and JUND proteins (*SI Appendix*, Fig. S7*D*), the latter a dimerization partner of FOS (69). Accordingly, increased luciferase activity was observed in BASP1-expressing cells after introducing an AP-1 responsive promoter construct (70) (*SI Appendix*, Fig. S7*E*). Furthermore, BASP1 activation leads to higher amounts of phosphorylated MAPK1 (ERK2) (*SI Appendix*, Fig. S7*C*), suggesting MAPK signaling promotion by BASP1 despite concomitant WNT pathway suppression.

### TNIK Inhibition Blocks MYC Expression and Reverts the Malignant Phenotype.

**Fig. 5.**
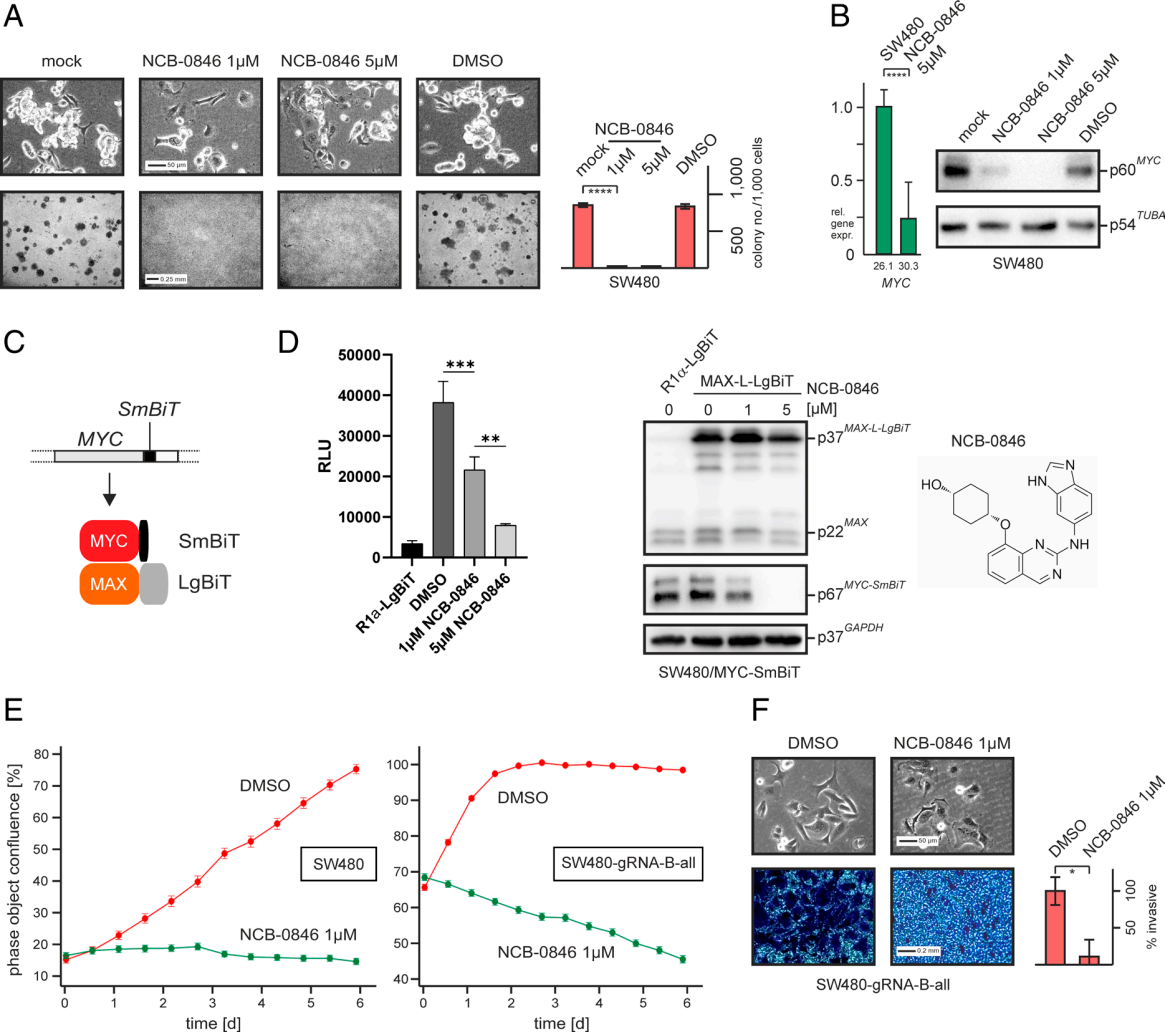
Pharmacological TNIK inhibition blocks MYC activity and BASP1-triggered cell invasion. (*A*) Cell morphology and colony formation in soft agar of SW480 cells after treatment with the compound NCB-0846 inhibiting TNIK (Traf2 and Nck-interacting kinase), which reverses the oncogenic phenotype. (*B*) MYC expression monitored at mRNA level by qPCR (*Left* panel) and protein level by Western blot analysis (*Right* panel). (*C*) Schematic depiction of the reporter system to measure endogenous MYC dimerization with MAX. (*D*) Impairment of MYC/MAX dimerization by NCB-0846 monitored by NanoBiT luciferase complementation assay. The cell line SW480/MYC-SmBiT containing a CRISPR-edited *MYC* allele, which encodes a MYC-small NanoBiT fusion protein, was transfected with the plasmid pcDNA3-MAX-L-LgBiT providing the large NanoBiT fragment fused to MAX. The construct pcDNA3-R1α-LgBiT containing the regulatory subunit of protein kinase A served as a negative control. *Left*: luciferase activities from cell extracts prepared two days after transfection (**P < 0.01, ***P < 0.001); middle: immunoblot analysis to monitor protein expression; *Right*: structural formula of NCB-0846. (*E*) IncuCyte-monitored proliferation of SW480 and SW480-gRNA-B-all cells treated with 1 µM NCB-0846 or with the solvent DMSO. (*F*) Invasion assay of SW480-gRNA-all cells treated with 1 µM NCB-0846 or DMSO. Cells passing the extracellular matrix were stained and quantified using the program ImageJ. Vertical bars show SE of the mean (SEM) from counted quadruplicates. Statistical significance was assessed by using an unpaired *t* test (*P < 0.05).

As shown above, the WNT signaling-associated protein kinase TNIK is downregulated upon genetic *BASP1* reactivation (Fig. 4*E*). TNIK phosphorylates the transcription regulator TCF7L2, a posttranslational modification that is required for its transcription-regulatory activity (60). To investigate the effect of pharmacological TNIK inhibition, the preclinical compound NCB-0846 was added to SW480 cells at low micromolar concentrations. The benzimidazole-aminoquinoline derivate NCB-0846 is an orally active and selective WNT signaling inhibitor that inactivates TNIK and abrogates colorectal cancer stemness (71) supporting the relevance of TNIK inhibitors for cancer treatment (72). NCB-0846-treated SW480 showed a significant morphological change towards a nonmalignant phenotype featured by a loss of anchorage-independent growth (Fig. 5*A*) although retaining their viability. Furthermore, a significant reduction of *MYC* mRNA and protein expression was observed at a 5-µM inhibitor concentration (Fig. 5*B*). To test for a direct impact on MYC activity, NCB-0846 was administered to SW480/MYC-SmBiT, which had been recently generated in our laboratory. This cell line contains a genetically modified *MYC* allele encoding a MYC protein fused carboxyl-terminally with the small 1.3 kDa-fragment of the NanoBiT luciferase (73). Upon transfecting SW480/MYC-SmBiT cells with the plasmid pcDNA3-MAX-L-LgBiT, which encodes MAX fused to the large 18-kDa NanoBiT fragment, NanoBiT luciferase is reconstituted due to MYC/MAX dimerization, allowing quantification of MYC activity in living cells (Fig. 5*C*). NCB-0846 treatment led to strong reduction of MYC-SmBiT protein levels and consequently to a loss of luciferase activity (Fig. 5*D*). These results show that TNIK represents a key protein kinase required for MYC activation in colon cancer cells, which was efficiently inhibited by a small molecule in agreement with previous data (71). Furthermore, these results demonstrate that pharmacological TNIK inhibition had a similar impact on transcriptional *MYC* activation as overexpressed BASP1 (*SI Appendix*, Fig. S8). To block residual TNIK activity in BASP1-expressing cells, NCB-0846 was added also to SW480-gRNA-B-all cells. Strikingly, this treatment led to a stronger cell proliferation arrest in comparison with SW480 (Fig. 5*E*). Moreover, this compound efficiently interfered with BASP1-triggered cell invasion (Fig. 5*F*) suggesting that in combination with genetic *BASP1* activation, TNIK inhibitors may represent valuable drugs to abrogate colon cancer oncogenicity.

## Discussion

Considering the eminent role of MYC in most human tumors, it is not surprising that numerous attempts have been made to interfere with the oncogenic activities of this major cancer driver. Due to its nonenzymatic biochemical functions and largely unstructured surface, MYC has been challenging to target with specific inhibitor compounds (74), and consequently, alternative attempts aiming indirect MYC inhibition have evolved (75). An indirect approach is to overexpress or to reactivate silenced genes in cancer cells, which normally counteract MYC such as *BASP1* (11). In the present study, a genetic system was employed based on recent investigations to reactivate dormant tumor suppressors (76), and as an appropriate human model system, colorectal cancer cells were selected, where *MYC* is activated through WNT/β-catenin signaling (4, 5). Overexpressing or reactivating *BASP1* not only led to *MYC* downregulation (cf. Fig. 2) but also to inhibition of the transformed phenotype in terms of anchorage-independent growth, contact inhibition, and tumor formation (cf. Fig. 3). Although protein levels of ectopic and endogenously activated BASP1 were comparable (cf. Fig. 2), cells with endogenously activated *BASP1* displayed a more pronounced effect in morphology and biological properties (cf. Fig. 3) including stronger transcriptional suppression of *MYC* resulting in significantly lower MYC protein levels (cf. Fig. 2). BASP1 activation and MYC repression are accompanied by downregulation of WNT pathway-associated genes and functional inhibition of this signaling pathway (cf. Fig. 4). This is in accordance with a recent study showing that BASP1 suppresses cell growth and metastasis through inhibiting the WNT/β-catenin pathway in gastric cancer (24). WNT signaling also regulates cellular metabolism in tumors, thereby facilitating lactate secretion (77) in agreement with lower lactate levels in BASP1-expressing SW480 (cf. Fig. 1).

Nuclear BASP1 has been described as transcriptional cosuppressor converting the Wilms tumor protein (WT1) from an oncoprotein into a tumor suppressor (13, 78). Oncogenic and tumor suppressive activities of WT1 are modulated by nuclear BASP1 upon physical interaction (13, 79), and BASP1-mediated WT1-repression is further relevant during reprogramming of differentiated cells into induced pluripotent stem cells (16). During epithelial–mesenchymal or mesenchymal–epithelial transition, WT1 either recruits CBP and p300 as coactivators or BASP1 as corepressor, respectively (80). Transcriptional repression is mediated by binding of BASP1 to phosphatidylinositol 4,5-bisphosphate (PIP2) and histone deacetylase (HDAC) (51, 79). The WT1/BASP1 complex diverts the differentiation program of the myelogenous leukemia cell line K562 toward a neuronal-like morphology exhibiting extensive arborization and expression of genes involved in neurite outgrowth and synapse formation (14). This is reminiscent of colon cancer cells overexpressing BASP1, which also exhibit enhanced expression of genes required for cell migration, axon guidance, neuronal development, and differentiation (cf. *SI Appendix*, Fig. S6).

Almost two thousand genes show significant differential regulation in the presence of activated BASP1 (cf. Fig. 4), suggesting that multiple responsive genes execute the transcriptional BASP1 program, apart from phenotypical changes induced by MYC downregulation. In fact, BASP1-induced phenotypical changes in SW480 are different from those obtained upon treatment with a chemical MYC inhibitor (81), indicating that activating dormant *BASP1* is not a mere substitute for direct MYC inhibition. This may be due to the pleiotropic BASP1 functions not only influencing MYC but also other transcription factors such as WT1 (15) or AP-1 (cf. *SI Appendix*, Fig. S7). On the other hand, one may ask if MYC known as an universal amplifier of gene expression (82) could have an influence on the almost thousand genes transcriptionally activated by BASP1 (cf. Fig. 4). We think that such an impact is rather low because MYC is significantly downregulated in BASP1-expressing cells, thus mitigating potential transcriptional amplification, which requires high-level MYC (82). Another important aspect in this context is that BASP1 interferes with v-Myc-induced cell transformation in the avian cell system where the v-*myc* oncogene is constitutively expressed from a retroviral promoter (10). Likewise, BASP1 overexpression induces significant morphological changes in avian fibroblasts probably resulting from a specific transcriptional program interfering with oncogenic v-Myc functions. In addition to that, BASP1 destabilizes the v-Myc protein by sequestering its interaction partner calmodulin (11). Upon challenging with BASP1 overexpression, these mechanisms acting on gene and protein levels could be also relevant for human cancer cells in which the *MYC* proto-oncogene is constitutively active due to chromosomal translocation or gene amplification.

In addition to its established function as a transcriptional repressor (13, 50, 83), BASP1 also mediates transcriptional activation as suggested by our transcriptomics data (cf. Fig. 4). For example, the *KISS1* gene is highly induced in the presence of BASP1, presumably upon direct promoter activation (cf. Fig. 4). In melanoma cells, where activated WNT signaling mediates cell motility and EMT, KISS1 is inhibited otherwise preventing colonization of disseminated cancer cells in distinct organs and the formation of secondary tumor foci (84). Hence, KISS1 is classified as metastasis suppressor with its expression commonly downregulated in relevant tumors of various origins. Conversely, additional proteins promoting metastasis progression are downregulated in BASP1-expressing cells like MTA1 or KAP1 (cf. Fig. 4). Malignant progression by MTA1 was found in breast cancer cells (38), and for KAP1 is known that it contributes to tumorigenesis in the same cancer type (61). Furthermore, KAP1 stabilizes mRNA of the *MYC* paralogue *MYCN,* thereby promoting neuroblastoma tumorigenicity (85). Another particular BASP1 target identified here encodes Traf2 and Nck-interacting kinase (TNIK), a serine/threonine kinase implicated in oncogenic WNT signaling, particularly in colorectal cancer, thereby promoting pro-tumorigenic functions that drive cell invasion and cancer stemness. TNIK-catalyzed phosphorylation activates TCF7L2 and is therefore required for transcriptional *MYC* induction in colon cancer cells (86). Pharmacological TNIK inhibition in SW480 cells using the compound NCB-0846 inhibits *MYC* transcription like overexpressed BASP1 does (cf. Fig. 5 and *SI Appendix*, Fig. S8), although BASP1 appears to trigger multiple additional processes such as enhanced cell migration or differentiation. Intriguingly, combining endogenous *BASP1* activation and TNIK repression resulted into abrogation of all cell transforming parameters in colon cancer cells including clonogenicity and invasiveness (cf. Fig. 5). This may enable future treatment options once appropriate TNIK inhibitors have surpassed ongoing clinical trial phases (87). Application of such small molecules would be advantageous due to favorable pharmacological properties (88) and because growth arrest and MYC inactivation was achieved without toxic side effects (cf. Fig. 5).

Canonical WNT/β-catenin signaling regulates cell pluripotency and determines the differentiation fate of cells during embryonic development and other physiological processes, but also drives pathogenic processes in colorectal cancer (89). These cancers are thus characterized by increased cell plasticity upon spreading through the body accompanied by differentiation to colonize diverse microenvironments (90, 91). Cellular activities such as migration, invasion, and colonization of distinct organs involve the expression of proteins important for adhesion like integrins and cadherins, proteins which are differentially expressed in BASP1-expressing cells (cf. *SI Appendix*, Fig. S3, and S6). Remarkably, BASP1 increases the migratory, invasive, and angiogenic potential of the investigated colon cancer cells (cf. *SI Appendix* Fig. 3, S5), which could result from a physiological BASP1 function as it may occur during neurogenesis, wound healing, or tissue regeneration. On the other site, these properties found in rare tumors evolved from BASP1-expressing SW480 cells could explain why in occasional human cancer types a tumor-promoting function of BASP1 has been reported. Nevertheless, in colon cancer cells featured by typical migratory, invasive, and metastatic signaling pathways (37, 59, 92), BASP1 blocks oncogenic WNT signaling and induces a tumor-suppressive and antimetastatic gene expression signature (cf. Figs. 1 and 4).

In most cases, high WNT activity correlates with concomitant MAPK signaling activation, which is required for tumor-initiation and modification of intratumoral heterogeneity (68). Interestingly, in BASP1-expressing colon cancer cells, the WNT pathway is inhibited (cf. Fig. 4), whereas MAPK signaling is not affected. On the contrary, moderate stimulation of this pathway was observed, including specific activation of the *FOS* gene (cf. *SI Appendix*, Fig. S7). Although *FOS* was initially defined as potent oncogene (69), an additional tumor-suppressive function has been recently reported in prostate cancer (93). This could be in line with our observations and corroborate a principal tumor-suppressive function of BASP1, which has been so far attributed to the majority of human cancer types in which this neuronal signaling protein is implicated.

## Materials and Methods

### Cells, Viruses, and Gene Transfer.

The large intestine hypotriploid cell line SW480 from Dukes C colorectal cancer (CCL-228) and its isogenic metastatic derivative SW620 (CCL-227), the breast cancer cell line MCF7 (HTB-22), and human embryonic kidney 293 cells (large T-antigen) (CRL-3216) were obtained from the American Type Culture Collection (ATCC, Manassas, VA, USA). SW480 and SW620 were cultivated using the original Leibovitz L-15 medium with no additional CO_2_, or in Dulbecco’s Modified Eagle Medium (DMEM), each supplemented with 10% (v/v) FBS in a water-saturated atmosphere at 5% CO_2_ (11). The latter medium was also used to cultivate K562 or HEK293T. Cells were kept *Mycoplasma*-free, which was routinely controlled by PCR (*SI Appendix*, Table S1). All cell lines had a passage number < 20 at the time of the respective experiment. Generation of the cell lines SW480-V, SW480-B, SW480-Bi, SW480-gRNA-B1, SW480-gRNA-B3, SW480-gRNA-B-all, SW480/MYC-SmBiT, SW620-B, and MCF7/BASP1-ko is described in the *SI Appendix*.

### Cell Biological Assays.

Cell proliferation was monitored in real time by using the live-cell imaging system IncuCyte S3 (Essen Bioscience/Sartorius, Vienna, Austria) as described (11), or upon dissociation in 0.25% trypsin-EDTA and cell number determination using a Coulter Counter (Beckman Coulter, Brea, CA, USA). To monitor cell migration in a scratch assay, each 20,000 cells were plated into wells of an IncuCyte Imagelock 96-well Microplate (BA-04855). When cells reached 80% confluency, a scratch was performed in each well using the IncuCyte cell migration kit (BA-04858). Cells were then analyzed with the IncuCyte scratch wound analysis software module and quantified. Agar colony and focus assays were performed as described (11). Invasion through an extracellular matrix (ECM) was determined using the ECM550 cell invasion assay kit (Merck, Darmstadt, Germany). Each 300,000 cells suspended in 300 µl serum-free DMEM were added into the upper invasion chamber containing a rehydrated ECM. The assembly was subsequently placed onto the lower chamber, consisting of an MP24 well filled with 500 µl DMEM containing 10% (v/v) FBS. After 72 h incubation at 37 °C, cells having passed the ECM were stained with eosin methylene blue and quantified using the program Image-Quant TL (GE Healthcare, Vienna, Austria). All biological assays were performed in n ≥ 3 replicates.

### Chorioallantoic Membrane (CAM) Assay.

Ex ovo CAM assays were conducted in agreement with ethical standards to national and international guidelines approved by the local Ethical Committee. Fertilized Lohmann chicken eggs (Schropper GmbH, Gloggnitz, Austria) were incubated for 3 d at 37.5 °C and 40 to 60% humidity in a rotary thermostat (FIEM snc, Guanzate, Italy). Eggs were opened using a portable drill and contents transferred into sterilized plastic weigh boats covered with square Petri dishes as described (55). After incubation for 6 d in a stationary incubator at 37.5 °C/75% humidity, each 3 autoclaved silicon rings with 5 mm diameter were placed onto the embryo CAM as described (94). Then, each 20 µl of a mixture consisting of 500,000 cells suspended in phosphate-buffered saline and 5 µl Matrigel matrix (Corning Inc, New York) was pipetted into the silicon rings. After 4-d incubation, developed tumors were scored, analyzed, and then embryos were sacrificed by hibernation. For tumor cell isolation, tumors were minced with a scalpel, and then cells were dissociated in 0.25% trypsin-EDTA for 30 min at 37 °C.

### RNA Analysis and Transcriptomics.

Total RNA isolation, poly(A)^+^-RNA selection, and Northern analysis was done as described (10, 95) using each 20 µg of total RNA and PCR-amplified digoxigenin-labeled DNA probes specific for the human *BASP1*, *MYC*, *MAX*, and *GAPDH* genes (*SI Appendix*, Table S1). Quantitative real-time PCR (qPCR) was carried out after cDNA synthesis from 100 ng 1× poly(A)^+^-selected RNA as described (95) using primer pairs specific for the human *BASP1*, *BASP1-AS1*, *MYC*, *TNIK*, *KISS1*, and *GAPDH* transcripts (*SI Appendix*, Table S1). To map the transcription start site of reactivated *BASP1* mRNA, 5’-rapid amplification of cDNA ends (RACE) was performed as described (10) with a kit (03353621001, Roche/Merck, Darmstadt, Germany) using 400 ng of poly(A)^+^-selected RNA from SW480-gRNA-B1 cells and human *BASP1*-specific oligodeoxynucleotides (*SI Appendix*, Table S1). Transcriptome determination (RNA-Seq) and analysis is outlined in the *SI Appendix*.

### Mass Spectrometry, Proteomics, and Metabolomics.

For proteome and metabolome analysis of the SW480-B and SW480-V cell lines, proteins, and metabolites were obtained from the same sample using simultaneous proteo-metabolome liquid–liquid extraction (SPM-LLE), with the protein interphase being dissolved in the urea-containing buffer as described (43). For proteomics, sample purification by reversed phase solid phase extraction (RP-SPE) and consecutive proteome analysis by LC–MS/MS was performed using a nanoUHPLC System (Dionex UltiMate 3000 RSLCnano pro flow, Thermo Fisher Scientific) coupled to an orbitrap tribrid mass spectrometer (Orbitrap Fusion Lumos, Thermo Fisher Scientific) as described (43). LC–MS/MS raw data were processed using Thermo Scientific Proteome Discoverer 2.4 SP1. For metabolomics, samples were analyzed using an ion chromatography HPLC (ICS-6000, Thermo Fisher Scientific) coupled to a quadrupole orbitrap (Orbitrap HF-X, Thermo Fisher Scientific), and raw data were processed using TraceFinder 5.0 (Thermo Fisher Scientific) as described (43). Post processing, statistics (Dixon’s Q test, Wilcoxon rank-sum test) and data visualization were carried out in R and RStudio using R package rstatix (https://cran.r-project.org/web/packages/rstatix/index.html) and ggplot2 (https://cran.r-project.org/web/packages/ggplot2/index.html). The LC–MS data that support the findings of this study are openly available at the following URL/DOI: https://doi.org/10.48323/0e4ra-wf143.

*Protein analysis, chromatin immunoprecipitation, and reporter assays.* See *SI Appendix*.

## Supplementary Material

Appendix 01 (PDF)

## Data Availability

RNA-Seq|LC-MS data have been deposited in Gene Expression Omnibus (NCBI)|Data Repository UIBK (GSE310401|https://doi.org/10.48323/0e4ra-wf143) (96, 97). All study data are included in the article and/or *SI Appendix*.
