# Supplementary material for: Reactivation of the silenced BASP1 gene suppresses oncogenic WNT signaling in human colorectal cancer cells
Source: Proc Natl Acad Sci U S A. 2026 Mar 5;123(10):e2524159123. doi: 10.1073/pnas.2524159123 (PMC12974518; doi:10.1073/pnas.2524159123)
Supplement: Supplementary file 1 — Appendix 01 (PDF) [file pnas.2524159123.sapp.pdf]

Revised manuscript (tracking #: 2025-24159)

PNAS

Supplementary information for

Reactivation of the silenced *BASP1* gene suppresses oncogenic WNT signaling in human colorectal cancer cells

Leonie I. Weber, Lea E. Timpen, Anna-Sophia Egger-Hörschinger, Philemon Schöpf, Nesin D. Ayhan, David Demmel, Madlen Hotze, Yang Zhang, Mahdi Mehrabi, Kane Puglisi, Eduard Stefan, Nassim Ghaffari-Tabrizi-Wizsy, José M. Ramos-Pittol, Marcel Kwiatkowski, Markus Hartl*

*Corresponding author
Email: markus.hartl@uibk.ac.at

This PDF file includes:

SI Materials and Methods
Tables 1-2
Figures S1-S8

Materials and Methods

Data base analyses and bioinformatics. Tumor-representative cell lines based on mRNA expression ratios of *MYC* and *BASP1* in human cancer cell lines and patient data were compared. For this reason, the computational tool *rna*Ratio was developed to select tumor-representative cell lines based on the mRNA expression ratios of genes to be compared. *rna*Ratio represents a package of the programming language R using the cBioPortal (1) R client termed cBioPortalData (2) to obtain RNA-Seq mRNA expression data from The Cancer Genome Atlas (TCGA) for both cell lines and patient cohorts. The program fetches expression data based on a user-given list of patient studies and a set of genes to be investigated. Thereby, patient studies are supplied either as TCGA study IDs or URLs of cBioPortal queries, whereas cell line screening studies are automatically included. For each data point, the expression value ratio is calculated, and then the data grouped and filtered by a set of cancer types given by the user. The selection of studies, cancer types, and genes to be investigated by *rna*Ratio is configurable by the user. Here, mRNA expression data of *MYC* and *BASP1* and mRNA expression ratios were calculated and visualized. The package source code is available via the Git hub at https://github.com/phschoepf/rnaRatio.

Gene annotation and enrichment analysis based on proteomic data were performed using the biomaRt (3, 4), clusterProfiler (5), and ggplot24 packages in R 4.4.15 (https://www.r-project.org/). Gene Ontology (GO) enrichment analysis was conducted using the clusterProfiler package to identify overrepresented biological processes (BP) among the dysregulated proteins. The analysis was performed using the enrichGO function, with human gene annotations from the org.Hs.eg.db database and gene symbols as input. Enrichment criteria included a minimum gene set size of 100, a maximum of 500, and statistical thresholds of p-value < 0.05 and q-value < 0.2 (Benjamini-Hochberg correction). The results were visualized using the dotplot function, with the top 30 enriched biological processes displayed.

Cells, viruses, and gene transfer. To generate SW480-V, SW480-B, or the BASP1-inducible SW480-Bi cell line, SW480 cells were nucleofected with the empty pcDNA3 vector (Invitrogen/Thermo Fisher Scientific, Waltham, MA, USA), pcDNA3-BASP1, or pAK-Tol2-TRE-BASP1 as described (6) using the cell line nucleofector kit V (Lonza, Basel, Switzerland) and the program L-24. Likewise, SW620-B were generated after nucleofection of pcDNA3-BASP1. To construct the BASP1 expression vectors, the PCR-amplified coding sequence of human *BASP1* was inserted into pcDNA3 containing a cytomegalovirus (CMV) promoter, or into pAK-Tol2-TRE-Puro containing a doxycycline-inducible promoter. pAK_Tol2_TRE_Puro was a gift from Michael Yaffe (Addgene plasmid # 130259; http://n2t.net/addgene:130259; RRID:Addgene_130259). Before single cell cloning, mass cultures of the transfected cells were analyzed by immunoblotting to confirm BASP1 expression. These cells were then selected with geneticin (G418) (1 mg/ml), or puromycin (1 µg/ml), respectively, and surviving cells subjected to single cell cloning in 96-well plates using appropriate dilutions. Constitutive and doxycycline-induced (1 µg/ml) BASP1-expressing cell clones were first verified by immunoblot analysis. Significant strong BASP1 expression was observed in 6 of 24 (SW480-B), 2 of 12 (SW480-Bi +dox), and 4 of 12 (SW620-B) analyzed cell clones, which were then expanded into the relevant cell lines.

CRISPR (clustered regularly interspaced short palindromic repeats)-based techniques were used for transcriptional *BASP1* activation, *BASP1* gene knock-out, and endogenous MYC protein tagging. For oligodeoxynucleotide design encoding small guide (sg) RNAs (SI, Table 1), the programs e-crisp (e-crispr.org) and chopchop (chopchop.cbu.uib.no) were used. Initially, four different sgRNAs were designed to reactivate the *BASP1* gene. Corresponding double-stranded oligodeoxynucleotides were inserted into the lenti sgRNA(MS2)-puro plasmid using the two *Bsm*BI restriction enzyme sites. lenti sgRNA(MS2)_puro optimized backbone was a gift from Feng Zhang (Addgene plasmid # 73797; http://n2t.net/addgene:73797; RRID:Addgene_73797). SW480-derived lines with reactivated endogenous *BASP1* (SW480-gRNA-B1, -B3, -B-all) were generated by three lentiviral transduction steps after each viral stock preparation. For this, 10 µg of the relevant lentiviral vector and each 5 µg of pMD2.G and psPAX2 were transfected into sub-confluent HEK293T cells grown on a 100-mm dish using the transfection reagent TransIT-LT1 (mirus/Merck, Darmstadt, Germany). pMD2.G and psPAX2 were gifts from Didier Trono (Addgene plasmid # 12259; http://n2t.net/addgene:12259; RRID:Addgene_12259, and Addgene plasmid # 12260;

http://n2t.net/addgene:12260; RRID:Addgene_12260, respectively). Origin and cultivation conditions of HEK293T cells have been described (7). After 48 h, lentivirus was harvested, filtrated using a polyvinylidene fluoride (PVDF) filter with 0.45 µM pore size, and subjected to ultracentrifugation at 16,500 × g for 2.5 h at 4 °C. For viral transduction, polybrene (hexadimethrine bromide, H9268, Sigma-Aldrich, St. Louis, MO, USA) was added to the filtered lentiviral medium (8 µg/ml). Transduction of SW480 cells was performed via spinoculation (8) at 700 × g for 45 min at 22 °C. After 48 h, cells were selected with respective antibiotics. SW480 cells were first transduced with virus derived from lenti MS2-P65-HSF1-Hygro. lenti MS2-P65-HSF1_Hygro was a gift from Feng Zhang (Addgene plasmid # 61426; http://n2t.net/addgene:61426; RRID:Addgene_61426) (8). After hygromycin B selection (600 µg/ml), the resulting cell line termed SW480-MS2 was transduced with virus derived from dCas9-VP64-GFP. dCAS9-VP64_GFP was a gift from Feng Zhang (Addgene plasmid # 61422; http://n2t.net/addgene:61422; RRID:Addgene_61422). Cells were analyzed for GFP expression via flow cytometry, and single cell cloning was then performed on GFP-positive cells resulting in the cell line SW480-MS2-dCas9. These cells were subsequently transduced with virus derived from lenti sgRNA(MS2)-puro derivatives, encoding the *BASP1* promoter-specific sgRNAs. lenti sgRNA(MS2)_puro optimized backbone was a gift from Feng Zhang (Addgene plasmid # 73797; http://n2t.net/addgene:73797; RRID:Addgene_73797) (8). Thereby viruses containing individual or pooled sgRNA plasmids were applied. Transduced cells were selected with puromycin (1 µg/ml), characterized by immunoblot analysis to test for BASP1 expression, and then expanded into the final cell lines.

To inactivate the endogenous *BASP1* gene in MCF7 cells, double-stranded oligodeoxynucleotides encoding a sgRNA complementary to a region encompassing the BASP1 translation start codon (SI, Table 1) were inserted into the Cas9-encoding lentiCRISPRv2 plasmid via the two *Bsm*BI sites to yield the vector lenti-CRISPRv2-BASP1-ko. lentiCRISPR v2 was a gift from Feng Zhang (Addgene plasmid # 52961; http://n2t.net/addgene:52961; RRID:Addgene_52961) (8). The construct was designed to induce a double-strand break in the region encompassing the start codon (ATG). The plasmid was lipofected into MCF7 and cells selected with puromycin (1 µg/ml) resulting in the cell line MCF7/BASP1-ko. Dysfunctionality of the original *BASP1* coding region was verified by DNA sequencing (Microsynth, Balgach, Switzerland) of a 295-bp PCR fragment, amplified from genomic DNA using the Monarch Extraction Kit (New England Biolabs, Ipswich, MA, USA) and a specific primer pair (SI, Table 1), and by immunoblotting.

To edit the *MYC* gene by providing a NanoBiT tag, a double-stranded oligodeoxynucleotide, encoding a sgRNA complementary to the last six codons of the human *MYC* coding sequence (SI, Table 1) was inserted into lentiCRISPRv2 via the two *Bsm*BI sites to yield the vector lenti-CRISPRv2-MYC. This construct is designed to induce a double-strand break one base pair before the stop codon. The plasmid was nucleofected into SW480 together with two anti-sense donor plasmids encoding the tia1l gRNA and containing a Linker-SmBiT-P2A-HPT cassette in the reading frames -1 and -2, flanked by tia1l-gRNA recognition sites (cLap-L-SmBiT-P2A-HPT -1, cLap-L-SmBiT-P2A-HPT -2). The donors were generated by removing part from the original cassettes (9) (a gift of Prof. F. Schnütgen, Goethe University Frankfurt, Germany) using the restriction enzymes *Sal*I and *Sma*I, and replacing it with assembled double-stranded oligodeoxynucleotides encompassing the tia1l-Linker-SmBiT-P2A and a part of the HPT segment (SI, Table 1). After hygromycin B selection (600 µg/ml), genomic DNA was isolated from single cell clones and analyzed by PCR with primers flanking the *MYC* integration site of the small NanoBiT (SmBiT) cassette (SI, Table 1). Three cell lines could be established encoding a functional MYC-SmBiT protein, which was verified by DNA sequencing (Microsynth, Balgach, Switzerland), immunoblot analysis, and protein complementation assay.

Histochemical cell staining and immunohistochemistry (IHC). Tumors derived from SW480 or SW480-gRNA-B-all cells seeded onto chicken chorioallantoic membranes were documented microscopically after 3 days. Afterwards, tumors were excised and fixed overnight in 4% (w/v) paraformaldehyde. Samples were dehydrated through graded ethanol series, cleared with toluene, paraffin-embedded, sectioned (5 µm), and then either stained with hematoxylin/eosin (H&E) as described (10), or subjected to Ki-67 immunohistochemistry using the UltraVision Quanto

HRP/DAB system (Thermo Fisher Scientific, Waltham, MA, USA). Sections were counterstained with hematoxylin and imaged on a BX53 microscope (Olympus, Tokyo, Japan).

RNA analysis and transcriptomics. For transcriptome analysis, total RNA was isolated from SW480, SW480-B, and SW480-gRNA-B-all, and each three replicates subjected to next generation sequencing (NGS). RNA library preparation, amplification and Illumina NovaSeq 6000 sequencing (RNA-Seq) were performed by the company Microsynth (Balgach, Switzerland). In total, 728,539,558 demultiplexed reads were performed with a mean read length of 138 bp. Gene expression analysis was performed using the GRCh37 reference (grch37.ensembl.org/Homo_sapiens). Transcriptome data from RNA sequencing (RNA-Seq) were analyzed for differential expression (DE), gene set enrichment (GSEA), and gene ontology (GO) using the program RaNA-Seq (ranaseq.eu) (11). RNA sequencing data have been deposited in NCBI's Gene Expression Omnibus (12) and are accessible through GEO Series accession number GSE310401 (https://www.ncbi.nlm.nih.gov/geo/query/acc.cgi?acc=GSE310401).

Protein analysis. Chromatin immunoprecipitation (ChIP) and quantitative ChIP (qChIP) were done as described (6, 13) using magnetic Dynabeads protein G beads (Thermo Fisher Scientific, Waltham, MA, USA). Prior to usage, beads were washed twice in ChIP dilution buffer. For pre-clearing of 1 ml of lysate, 10 µl of bead suspension were used followed by immunoprecipitation using 40 µl bead suspension.

Co-immunoprecipitation (Co-IP) was performed as described (7) with following modifications. Each three 150-mm dishes of SW480 and SW480-gRNA-B-all cells were grown at 80% confluency. Cells were lysed in 2 ml of IP lysis buffer (40 mM HEPES pH 7.5, 120 mM NaCl, 1 mM EDTA, 0.3% CHAPS) containing protease and phosphatase inhibitors per dish. Pooled lysates were put onto a rotary shaker for 20 min at 4 °C. After centrifugation at 600 × g for 3 min at 4 °C, protein concentrations were measured using the Bradford protein assay dye reagent concentrate (Bio-Rad Laboratories, Watford, UK) and protein amounts adjusted accordingly. For preincubation, 10 µl of washed magnetic Dynabeads protein G beads were used per 1 ml of lysate. Preincubation was performed for 30 min on a rotary shaker at 4 °C, and then a 100-µl input was taken. Next, 7.5 µg of respective antibody was added per 1 ml lysate and incubated on a rotary shaker for 30 min at 4 °C. For precipitation, 37.5 µl of the washed beads were added to 1 ml of lysate and incubated on a rotary shaker for 1.5 h at 4 °C. After supernatant removal, beads were washed five times (last two washes for each 10 min) at 4 °C on a rotary shaker using IP lysis buffer. Beads were finally suspended in 40 µl of 1x protein gel electrophoresis (Laemmli) buffer. Prior to gel electrophoresis samples were heated to 70 °C for 10 min.

Protein pull-down was done as described (7) using purified recombinant GST-BASP1 protein encoded by the plasmid pET42a-BASP1. To construct this prokaryotic expression vector, a DNA fragment encompassing the human *BASP1* coding sequence from pAK-Tol2-TRE-Puro-BASP1 was inserted via the *Nco*I and *Sal*I sites into pET-42a(+) (Merck, Darmstadt, Germany).

Immunoblotting (IB) was done as described (7) using antibodies (SI, Table 2) specifically recognizing the analyzed human proteins. The immunoaffinity-purified antibody directed against a recombinant GST-BASP1 fusion protein (a gift of Prof. S. Roberts, Virginia Tech, USA) has been described (14). An immunopurified peptide antiserum directed against the conjugated peptide PVEAPAANSDQTVTVKE representing the last 17 carboxyterminal amino acid residues of the human BASP1 protein was generated by the company Biotrend (Cologne, Germany) as described previously (6).

Promoter and protein complementation analyses. Luciferase assays were done as described (7) after transfecting cells using Lipofectamine 3000 (Thermo Fisher Scientific, Waltham, MA, USA). To construct pLUC-MYC and pLUC-KISS1, relevant 1.9 kbp and 3.1 kbp promoter fragments, respectively, including the 5'-transcription start sites were amplified from SW480 genomic DNA and inserted via the *Kpn*I and *Hind*III sites of the firefly luciferase pGL3-Basic (Promega, Madison, WI, USA) reporter plasmid. To construct pLUC-BASP1, an 801-bp fragment from the human *BASP1* promoter including the first 80 bp from exon 1 was synthesized by Genscript Biotech (Rijswijk, Netherlands) and inserted into pGL3-Basic as described above. All constructs were verified by DNA sequencing (Microsynth, Balgach, Switzerland).

To monitor WNT signaling the TOP/FOP-Flash luciferase reporter assay was applied. Cells were seeded into wells of a 96-well plate transfected with 80 ng of the TCF reporter plasmid TOP, or FOP (15), and 40 ng of TK-Renilla plasmid E2241 (Promega, Madison, WI, USA), using Lipofectamine 3000 (Thermo Fisher Scientific, Waltham, MA, USA). Cells were cultivated in WNT3a-conditioned medium or control medium for 48 h. WNT3a-conditioned medium was prepared as described (16) from L-WNT3a cells (a gift of Prof. H. Clevers, Hubrecht Institute, Utrecht, The Netherlands), and mixed 1:1 with normal medium.

To measure AP-1 activity, the reporter pLUC-JAC was used where an 802-bp fragment of the chicken *JAC* promoter (17) has been inserted into pGL3-Basic (Promega, Madison, WI, USA). Cells were seeded into a 96-well plate and each well was transfected with 90 ng of the *JAC* reporter plasmid and 10 ng of the CMV-Renilla plasmid E2261 (Promega, Madison, WI, USA) using Lipofectamine 3000 (Thermo Fisher Scientific, Waltham, MA, USA). Promoter activity was determined using the Dual-luciferase reporter assay system (Promega, Madison, WI, USA). After washing cells with phosphate-buffered saline (PBS), each 20 µl of 1x Passive lysis buffer was added to each well, and the plate rocked at 600 rpm for 15 min at 22 °C. Lysates of each well were then transferred into a white 96 well plate. To each well, 50 µl of Luciferase assay substrate were added, and luminescence measured using the Thermo Scientific Varioskan LUX multimode microplate reader. After measurement, 50 µl of the Stop & Glo substrate were added and luminescence was measured again. The Firefly luciferase signal was normalized to the Renilla luciferase signal by calculating the ratio of Firefly/Renilla luciferase.

Protein complementation assay was carried out as described (18) with following modifications. Each 1 × 10^6 SW480/MYC-SmBiT cells were seeded into wells of an MP6 plate. The next day, cells were transfected with each 500 ng pcDNA3-MAX-L-LgBiT or pcDNA3-R1α-LgBiT using Lipofectamine 3000. After 6 h, NCB-0846 or the solvent dimethyl sulfoxide (DMSO) were added and cells incubated for 2 d. For luminescence measurement, cells were scraped in 150 µl phosphate-buffered saline (PBS) and transferred into wells of a 96-well plate. 20 µl of 1 mM benzyl-coelenterazine in PBS were added to each well and luminescence measured using the device PHERAstar FSX (BMG Labtech, Ortenberg, Germany). pcDNA3-MAX-L-LgBiT encodes the human MAX protein with carboxyl-terminally fused large fragment of NanoBiT luciferase (LgBiT) separated by a linker (L). To construct pcDNA3-MAX-L-LgBiT, the LgBiT-tag coding sequence was PCR-amplified from the vector pFC34K-LgBiT-TK-neo Flexi N2015 (Promega, Madison, WI, USA). The PCR product was then used to replace the RLuc1 segment from the pcDNA3-MAX-L-RLuc1 (18) vector, which encodes MAX and the linker GGGGSGGGS, using the restriction enzyme sites *Bsp*EI and *Xba*I. pcDNA3-R1α-LgBiT encodes the subunit R1α of human protein kinase A fused to LgBiT, which served as a negative control.

References

1. J. Gao *et al.*, Integrative analysis of complex cancer genomics and clinical profiles using the cBioPortal. *Sci Signal* **6**, pl1 (2013).
2. M. Ramos *et al.*, Multiomic Integration of Public Oncology Databases in Bioconductor. *JCO Clin Cancer Inform* **4**, 958-971 (2020).
3. S. Durinck *et al.*, BioMart and Bioconductor: a powerful link between biological databases and microarray data analysis. *Bioinformatics* **21**, 3439-3440 (2005).
4. S. Durinck, P. T. Spellman, E. Birney, W. Huber, Mapping identifiers for the integration of genomic datasets with the R/Bioconductor package biomaRt. *Nat Protoc* **4**, 1184-1191 (2009).
5. G. Yu, L. G. Wang, Y. Han, Q. Y. He, clusterProfiler: an R package for comparing biological themes among gene clusters. *OMICS* **16**, 284-287 (2012).
6. M. Hartl, A. Nist, M. I. Khan, T. Valovka, K. Bister, Inhibition of Myc-induced cell transformation by brain acid-soluble protein 1 (BASP1). *Proc Natl Acad Sci U S A* **106**, 5604-5609 (2009).
7. M. Hartl, K. Puglisi, A. Nist, P. Raffeiner, K. Bister, The brain acid-soluble protein 1 (BASP1) interferes with the oncogenic capacity of MYC and its binding to calmodulin. *Mol Oncol* 10.1002/1878-0261.12636 (2020).

8. N. E. Sanjana, O. Shalem, F. Zhang, Improved vectors and genome-wide libraries for CRISPR screening. *Nat Methods* **11**, 783-784 (2014).
9. F. M. B. Thone, N. S. Kurrle, H. von Melchner, F. Schnutgen, CRISPR/Cas9-mediated generic protein tagging in mammalian cells. *Methods* **164-165**, 59-66 (2019).
10. F. Lyssy *et al.*, The chicken chorioallantoic membrane assay revisited - A face-lifted approach for new perspectives in placenta research. *Placenta* **166**, 77-84 (2025).
11. C. Prieto, D. Barrios, RaNA-Seq: Interactive RNA-Seq analysis from FASTQ files to functional analysis. *Bioinformatics* 10.1093/bioinformatics/btz854 (2019).
12. R. Edgar, M. Domrachev, A. E. Lash, Gene Expression Omnibus: NCBI gene expression and hybridization array data repository. *Nucleic Acids Res* **30**, 207-210 (2002).
13. T. Valovka *et al.*, Transcriptional control of DNA replication licensing by Myc. *Sci Rep* **3**, 3444 (2013).
14. B. Carpenter *et al.*, BASP1 is a transcriptional cosuppressor for the Wilms' tumor suppressor protein WT1. *Mol Cell Biol* **24**, 537-549 (2004).
15. F. J. Staal, M. van Noort, G. J. Strous, H. C. Clevers, Wnt signals are transmitted through N-terminally dephosphorylated beta-catenin. *EMBO Rep* **3**, 63-68 (2002).
16. C. Pleguezuelos-Manzano *et al.*, Establishment and Culture of Human Intestinal Organoids Derived from Adult Stem Cells. *Curr Protoc Immunol* **130**, e106 (2020).
17. M. Hartl, F. Reiter, A. G. Bader, M. Castellazzi, K. Bister, JAC, a direct target of oncogenic transcription factor Jun, is involved in cell transformation and tumorigenesis. *Proc Natl Acad Sci U S A* **98**, 13601-13606 (2001).
18. P. Raffeiner *et al.*, In vivo quantification and perturbation of Myc-Max interactions and the impact on oncogenic potential. *Oncotarget* **5**, 8869-8878 (2014).
19. A. J. Moorhouse, A. E. Loats, K. F. Medler, S. G. E. Roberts, The BASP1 transcriptional corepressor modifies chromatin through lipid-dependent and lipid-independent mechanisms. *iScience* **25**, 104796 (2022).
20. C. Y. Ewald *et al.*, TNIK's emerging role in cancer, metabolism, and age-related diseases. *Trends Pharmacol Sci* **45**, 478-489 (2024).

Figures and Tables

Table 1 Applied oligodeoxynucleotides

Oligodeoxynucleotide (from 5' to 3')	Type	Purpose	Usage
GGGAGCAAACAGGATTAGATACCCT	sense	Mycoplasma test	PCR
TGCACCATCTGTCACTCTGTTAACCTC	antisense	Mycoplasma test	PCR
AATTCCATGGGAGGCAAGCTCAGC	sense	pcDNA3-BASP1	PCR
GATCGTCGACTCACTCTTTCACGGTTAC	antisense	pcDNA3-BASP1	PCR
CACCGGAGGAACCAGGATGGCGGAT	sense	*BASP1*-sgRNA1	CRISPRa
AAACATCCGCCATCCTGGTTCCTCC	antisense	*BASP1*-sgRNA1	CRISPRa
CACCGAGCGGAATCGTAGTCGACGT	sense	*BASP1*-sgRNA2	CRISPRa
AAACACGTCGACTACGATTCCGCTC	antisense	*BASP1*-sgRNA2	CRISPRa
CACCGGCATTTAGGAAACGTTGGCG	sense	*BASP1*-sgRNA3	CRISPRa
AAACCGCCAACGTTTCCTAAATGCC	antisense	*BASP1*-sgRNA3	CRISPRa
CACCGGCACTGGGCAGGAAGGGGAG	sense	*BASP1*-sgRNA4	CRISPRa
AAACCTCCCCTTCCTGCCCAGTGCC	antisense	*BASP1*-sgRNA4	CRISPRa
TTTTGGGTTCCCCTTCCTCCTTG	antisense	*BASP1*-1 5'RACE	PCR
CCTTCTTCTTCTTGCTGAGC	antisense	*BASP1*-2 5'RACE	PCR
CTATGCAGCCCCTGAGTTAG	antisense	*BASP1*-3 5'RACE	PCR
CACCGCTACGGAACTCTTGTGCGTA	sense	MYC-SmBiT	CRISPR tag
AAACTACGCACAAGAGTTCCGTAGC	antisense	MYC-SmBiT	CRISPR tag
TCGACTACCCTGGAGAGGTTCCCGACATACCCTGGTGG	assembly	L-SmBiT-P2A -1	Donor vector
CGGTGGCTCTGGAGGTGGTGGGTCCTCCGGAGTGACC GGCTACCG	assembly	L-SmBiT-P2A -1	Donor vector
AGAATCTCCTCGAACAGCCGGTAGCCGGTCACTCCGGA GGACCCA	assembly	L-SmBiT-P2A -1	Donor vector
CCACCTCCAGAGCCACCGCCACCAGGGTATGTCGGGA ACCTCTCCAGGGTAG	assembly	L-SmBiT-P2A -1	Donor vector
GCTGTTCGAGGAGATTCTGGCCACGAACTTCTCTCTGTT AAAGCAAGCAGGAGATGTTGAAGAAAACCCC	assembly	L-SmBiT-P2A -1	Donor vector
GGGGTTTTCTTCAACATCTCCTGCTTGCTTTAACAGAGA GAAGTTCGTGGCC	assembly	L-SmBiT-P2A -1	Donor vector
TCGACTACCCTGGAGAGGTTCCCGACATACCTGGTGGC	assembly	L-SmBiT-P2A -2	Donor vector
GGTGGCTCTGGAGGTGGTGGGTCCTCCGGAGTGACCG GCTACCGG	assembly	L-SmBiT-P2A -2	Donor vector
CAGAATCTCCTCGAACAGCCGGTAGCCGGTCACTCCGG AGGACCC	assembly	L-SmBiT-P2A -2	Donor vector
ACCACCTCCAGAGCCACCGCCACCAGGTATGTCGGGAA CCTCTCCAGGGTAG	assembly	L-SmBiT-P2A -2	Donor vector
CTGTTCGAGGAGATTCTGGCCACGAACTTCTCTCTGTTA AAGCAAGCAGGAGATGTTGAAGAAAACCCC	assembly	L-SmBiT-P2A -2	Donor vector
GGGGTTTTCTTCAACATCTCCTGCTTGCTTTAACAGAGA GAAGTTCGTGGC	assembly	L-SmBiT-P2A -2	Donor vector
TACCGGCTGTTCGAGGAGATTC	sense	MYC-SmBiT	Genomic PCR
AGTCCAATTTGAGGCAGTTTAC	antisense	MYC-SmBiT	Genomic PCR
CAGAGGAGCAAAAGCTCATTTC	sense	SmBiT-P2A-HPT	Genomic PCR
AATCTCCTCGAACAGCCGGTAG	antisense	SmBiT-P2A-HPT	Genomic PCR
AGTCTTCACACTCGAAGATTT	sense	pc.-MAX-L-LgBiT	PCR
CTATGTCTAGATTAGCTGTTGATGGTTACTCGGAA	antisense	pc.-MAX-L-LgBiT	PCR
CACCGTGCCTCCCATCTTGGAGTTC	sense	*BASP1*-ko sgRNA	CRISPR ko
AAACGAACTCCAAGATGGGAGGCAC	antisense	*BASP1*-ko sgRNA	CRISPR ko
TGGGTAACATAGCGAGACC	sense	*BASP1*-ko locus	Genomic PCR
CTCGGCCTTCTTGTCTTTC	antisense	*BASP1*-ko locus	Genomic PCR
GAGAAAGCCAAGGAGAAAGAC	sense	*BASP1*	PCR (DIG)
TTTTGGGTTCCCCTTCCTC	antisense	*BASP1*	PCR (DIG)
GAGGAACAAGAAGATGAGGAAG	sense	*MYC*	PCR (DIG)
TCACGCAGGGCAAAAAAG	antisense	*MYC*	PCR (DIG)
AATCTGCGGCTGACAAAC	sense	*MAX*	PCR (DIG)
GAGCTTCTTCCTGCTTTGG	antisense	*MAX*	PCR (DIG)
GAAATCCCATCACCATCTTCC	sense	*GAPDH*	PCR (DIG)
TGAGTCCTTCCACGATACC	antisense	*GAPDH*	PCR (DIG)
GAGGGAGCTGACTGATACACTC	sense	*FOS*	PCR (DIG)
CTCTTGACAGGTTCCACTGAGG	antisense	*FOS*	PCR (DIG)
GACCTTCTATGACGATGCCCTC	sense	*JUN*	PCR (DIG)
TGCTCATCTGTCACGTTCTTGG	antisense	*JUN*	PCR (DIG)

GTGAAAGAGTGACAAGGACAG	sense	*BASP1*	qPCR
AGGAGAGAGAGAGAGGAGAG	antisense	*BASP1*	qPCR
TCGACTACGATTCCGCTAC	sense	*BASP1-AS1*	qPCR
CCTTTCCCCTCTTCTCTTCC	antisense	*BASP1-AS1*	qPCR
AGTGGAAAACCAGCAGCCTC	sense	*MYC*	qPCR
TTCTCCTCCTCGTCGCAGTA	antisense	*MYC*	qPCR
AAGCATCCATTTATACGAGACC	sense	*TNIK*	qPCR
CTCCTCCTCTTCTTCCTCAC	antisense	*TNIK*	qPCR
TAGGACCTGCCTCTTCTCAC	sense	*KISS1*	qPCR
GCCACCTTTTCTAATGGCTC	antisense	*KISS1*	qPCR
ACAGTCAGCCGCATCTTCTT	sense	*GAPDH*	qPCR
ACGACCAAATCCGTTGACTC	antisense	*GAPDH*	qPCR
GGAGGGAGGTTTGTGAGAGC	sense	*JUN*	qPCR
ACAAACAACACTGGGCAGGA	antisense	*JUN*	qPCR
TGGTGGCCTCTCTCTACACGA	sense	*JUNB*	qPCR
GGGTCGGCCAGGTTGAC	antisense	*JUNB*	qPCR
AGCCTCAAACCCTGCCTTTC	sense	*JUND*	qPCR
ACACACACACACACAACCAAC	antisense	*JUND*	qPCR
GCATTACAGAGAGGAGAAACAC	sense	*FOS*	qPCR
AGAAAAGAGACACAGACCCAG	antisense	*FOS*	qPCR
ACTCTCCATCACCCTCTTCC	sense	*FOSL1*	qPCR
TCCGCAGATCAGCTCATCAC	antisense	*FOSL1*	qPCR
GAAAGAGGGAGAGAGAGAGAG	sense	*FOSL2*	qPCR
AAAAGGAGCCGGAGAATAAAC	antisense	*FOSL2*	qPCR
AACCTCTTCCTATCTCAGCTC	sense	*MYC* (s.-enh.)	ChIP
ACTTTTCTCAGTGCCTTTCATC	antisense	*MYC* (s.-enh.)	ChIP
GAAATTAATGCCTGGAAGGCAG	sense	*MYC* (dist.)	ChIP
TCTTTCCTCCCCGGACAAAC	antisense	*MYC* (dist.)	ChIP
TCTACACTAACATCCCACGCTC	sense	*MYC* (prox.)	ChIP
AATCATCGCAGGCGGAACAG	antisense	*MYC* (prox.)	ChIP
CGCCATTACAGCTTCAAATTG	sense	*MYC* (3'-w.r.e.)	ChIP
GCCTGGACTTAGTGATTCAGAC	antisense	*MYC* (3'-w.r.e.)	ChIP
GTAACCCGTTGAACCCCATT	sense	Ctrl.1 18S (19)	ChIP
CCATCCAATCGGTAGTAGCG	antisense	Ctrl.1 18S (19)	ChIP
CAGCTCAGTGCTGTTGGTGG	sense	Ctrl. 2 BAX (19)	ChIP
ACCATCCAACCCTGGAGATC	antisense	Ctrl. 2 BAX (19)	ChIP
CATGGGTACCTGGGCAACTAGCTAAGTCG	sense	pLUC-MYC	PCR
CATGAAGCTTAATGGGCAGAATAGCCTCC	antisense	pLUC-MYC	PCR
CATGGGTACCGAACACAGGAGTGCAGAATC	sense	pLUC-KISS1	PCR
CATGAAGCTTGCATCTGTCCGTCTTAGAAC	antisense	pLUC-KISS1	PCR

Table 2 Applied antibodies

**Antibody (h)**	**Order #**	**Provider**	**Origin**	**Purpose**	**Dilution**
anti-BASP1	gift	Stefan Roberts, Virginia Tech, USA	rabbit	IB	1:500
anti-BASP1-CT	customized	Biotrend, Cologne, Germany	rabbit	ChIP/CoIP	4.0 / 7.5 µg/ml
anti-CTNNB1	sc-7963	Santa Cruz, Dallas, TX, USA	mouse	IB	1:1,000
anti-ERK1/2-P	4370S	Cell Signaling, Danvas, MA, USA	rabbit	IB	1:1,000
anti-JUN	sc-74543	Santa Cruz, Dallas, TX, USA	mouse	IB	1:500
anti-JUNB	sc-8051	Santa Cruz, Dallas, TX, USA	mouse	IB	1:500
anti-JUND	sc-271938	Santa Cruz, Dallas, TX, USA	mouse	IB	1:500
anti-FOS	sc-166940	Santa Cruz, Dallas, TX, USA	mouse	IB	1:500
anti-FOSL1	5281	Cell Signaling, Danvas, MA, USA	rabbit	IB	1:1,000
anti-FOSL2	sc-166102	Santa Cruz, Dallas, TX, USA	mouse	IB	1:500
anti-GAPDH	#AB8245	Abcam, Cambridge, UK	mouse	IB	1:10,000
Anti-KAP1	4124	Cell Signaling, Danvas, MA, USA	rabbit	IB	1:1,000
anti-MAX	Sc-8011	Santa Cruz, Dallas, TX, USA	mouse	IB	1:200
anti-MTA1	sc-373765	Santa Cruz, Dallas, TX, USA	mouse	IB	1:500
anti MYC	D3N8F	Cell Signaling, Danvas, MA, USA	rabbit	IB	1:1,000
anti-PHB2	14085	Cell Signaling, Danvas, MA, USA	rabbit	IB	1:1,000
anti-STK4 (krs2)	sc-515051	Santa Cruz, Dallas, TX, USA	mouse	IB	1:500
anti-TCF7L2	sc-166699	Santa Cruz, Dallas, TX, USA	mouse	IB	1:500
anti-TNIK	sc-377215	Santa Cruz, Dallas, TX, USA	mouse	IB	1:500
anti-TUBA	T5168	Sigma-Aldrich, St. Louis, MO, USA	mouse	IB	1:10,000
anti-VCL	4650	Cell Signaling, Danvas, MA, USA	rabbit	IB	1:1,000
anti-Ki-67	GA626	Agilent/Dako, Vienna, Austria	mouse	IHC	1:100

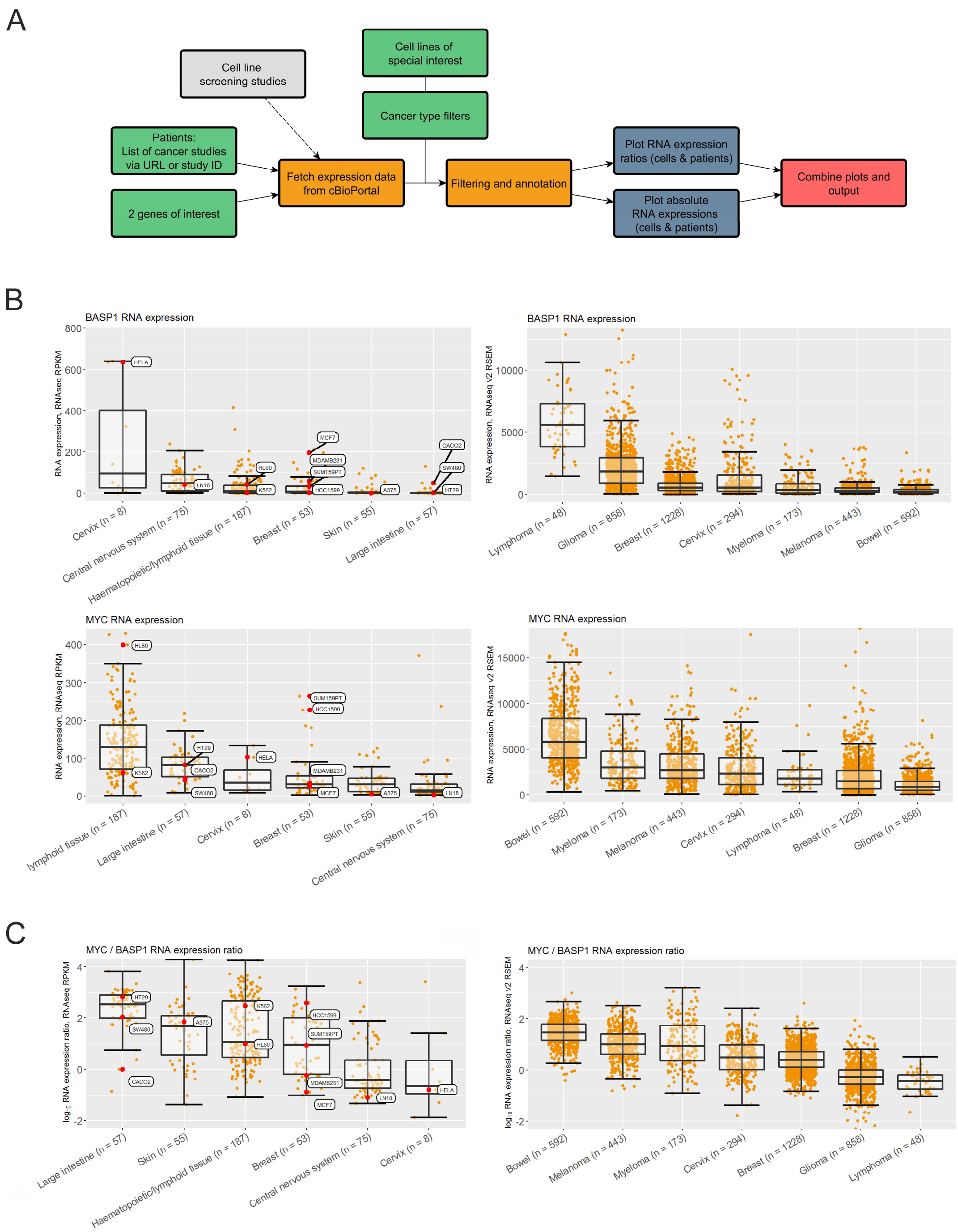

Figure S1. Development and application of the *rna*Ratio program to identify clinically relevant human cancer cell lines expressing high *MYC* and low *BASP1* levels. (A) Typical program workflow (green, user inputs; grey, static input data; orange, cBioPortal data fetching stage; blue, plotting stage; red, output). (B) Left panels: *BASP1* and *MYC* expression graphs in human cancer cell lines. Right panels: *BASP1* and *MYC* expression graphs in patient cohorts. (C) Expression ratio graphs. Left panel: *MYC*/*BASP1* mRNA expression ratio plot from permanent human cancer cells showing the positions of characteristic cell lines. The positions of representative cell lines (white boxes) are indicated by red dots (e.g. SW480 and MCF7 with higher or lower *MYC*/*BASP1* ratios, respectively).

Right panel: *MYC*/*BASP1* expression ratio plot from patient cohorts for correlation analysis. The highest *MYC*/*BASP1* expression ratio was found in cancer tissue derived from patients with bowel (intestine) cancers. The SW480 cell line is derived from a primary colon adenocarcinoma.

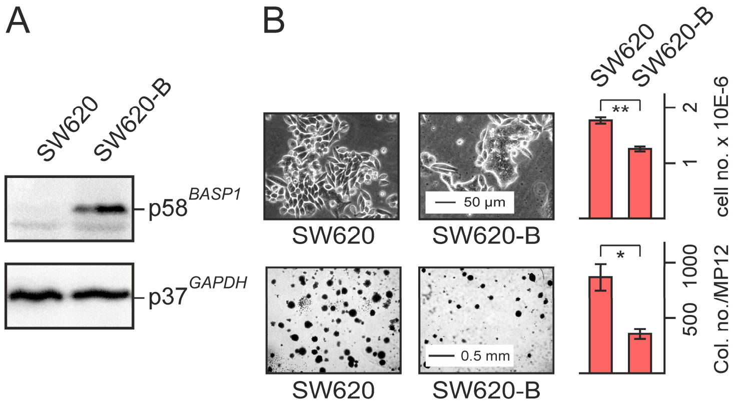

Figure S2. Ectopic BASP1 expression interferes with the transformed phenotype of SW620 cells, which are derived from metastatic colorectal cancer. SW620 cells were transfected with pcDNA3-BASP1 and a representative cell line (SW620-B) was obtained after geneticin (G418) selection. (A) Immunoblot analysis showing expression of ectopic BASP1 and of GAPDH, the latter used as control. (B) Ectopic human BASP1 causes morphological changes and reduced proliferation (micrographs of cells at the top) and an impaired transformed phenotype (colony formation in soft agar at the bottom). Vertical bars show standard deviations (SD) from independent experiments. Statistical significance was assessed by using an unpaired Student t-test (*$P < 0.05$, **$P < 0.01$).

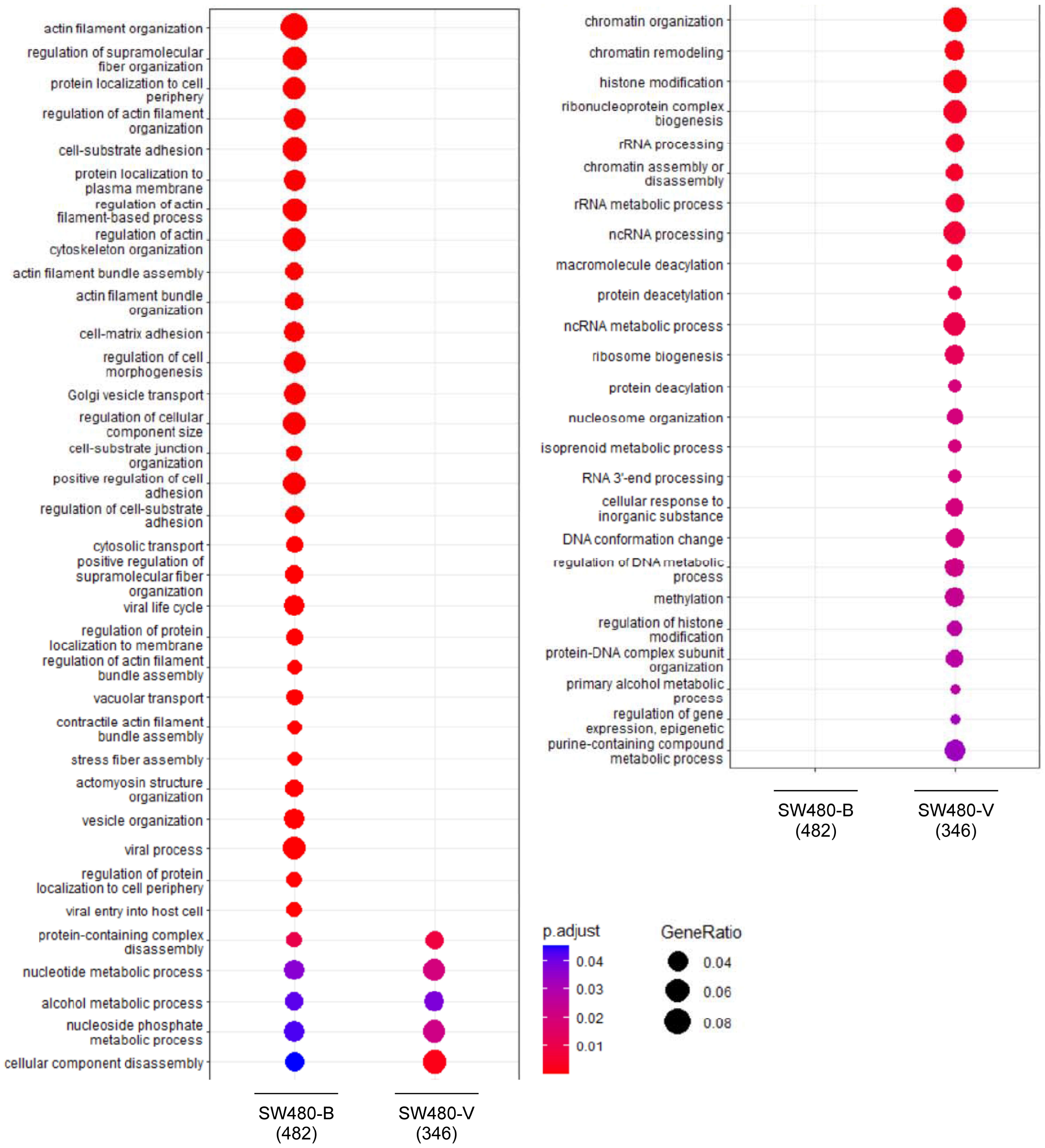

Figure S3. Comparison of SW480-B and SW480-V gene ontology (GO) analyses based on relevant proteome data. Proteins >2 fold overexpressed in SW480-B (482 proteins) or in SW480-V (346 proteins) were analyzed according to biological processes (BP), which are depicted as circles. The color reflects the p-value of the analysis, and the area is proportional to the gene ratio (portion of relevant genes within a cluster). Proteins specifically present in SW480-B are for instance involved in the regulation of actin filament polymerization, cell adhesion, or stress fiber assembly. Proteins specifically expressed in SW480-V are implicated in processes like gene regulation or chromatin assembly. Only five biological process clusters are shared between both cell types.

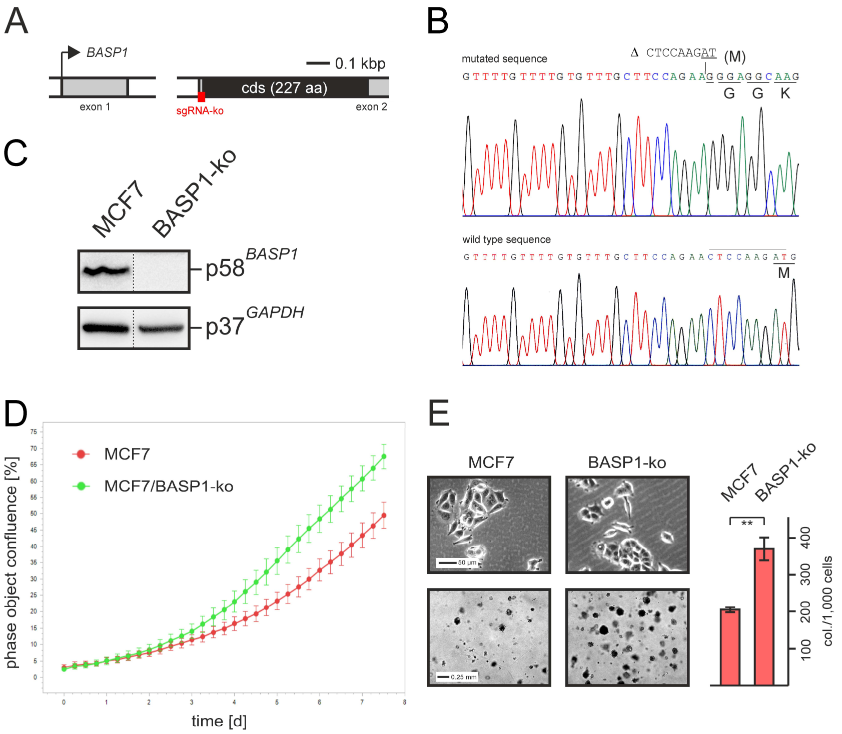

Figure S4. CRISPR-mediated inactivation of *BASP1* in MCF7 cells. (A) Schematic depiction of the human *BASP1* gene with the sgRNA binding site position shown as red bar (sgRNA-ko). (B) Genomic sequence context of a *BASP1* region showing the 9-bp deletion (Δ), which prevents initiation of BASP1 protein translation due to the lack of a functional start codon ATG encoding methionine (M). The sequence of the corresponding DNA segment from MCF7 (wild type) with the intact start codon is shown below. (C) Immunoblot analyses of the MCF7/BASP1-ko cell line in comparison with parental MCF7. The dotted lines mark splicing sites in the blots, from which one redundant lane has been removed. (D) IncuCyte-monitored proliferation of MCF7 and MCF7/BASP1-ko cells. (E) Cell morphologies and colony formation in soft agar of MCF7 and of MCF7/BASP1-ko cells. Vertical bars show standard deviations (SD) from independent experiments. Statistical significance was assessed by using an unpaired Student t-test (**P < 0.01).

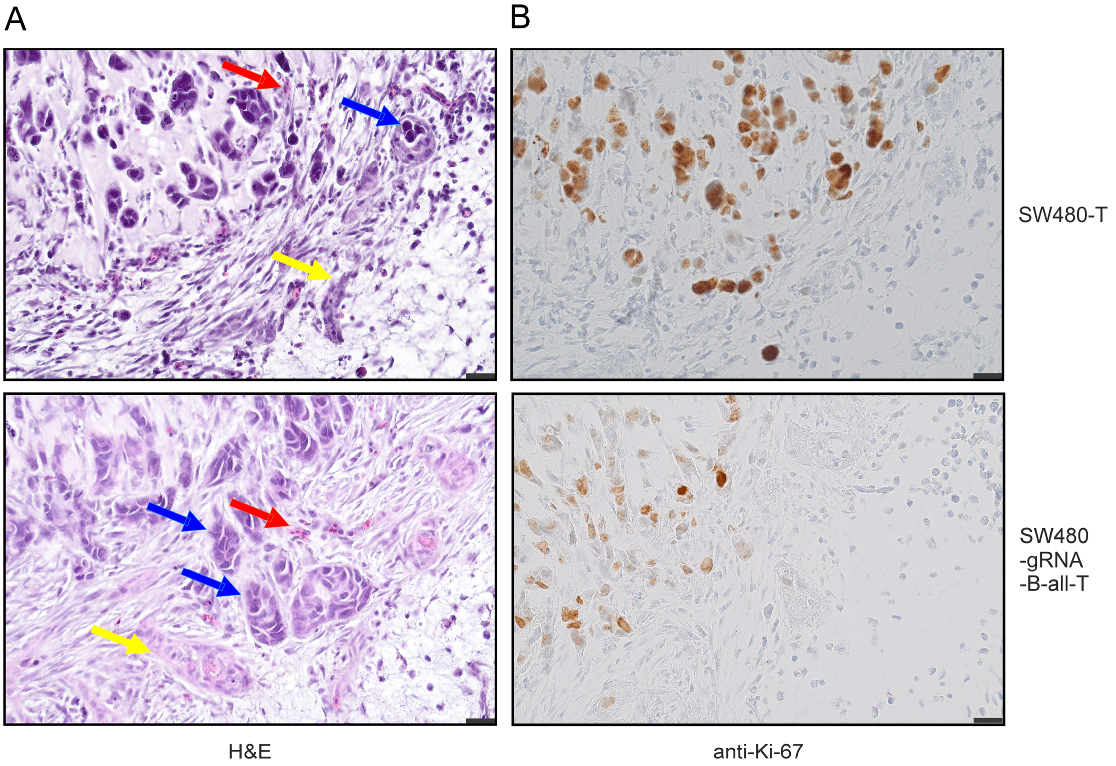

Figure S5. Representative comparison of tumor (T) sections derived from SW480 and SW480-gRNA-B-all cells seeded onto chicken chorioallantoic membranes (CAM). (A) Histochemical hematoxylin and eosin (H&E) staining. Red arrows show angiogenesis, yellow arrows mark invaded CAM epithelium, and blue arrows highlight invasive tumor cells indicating that SW480-gRNA-B-all-T cells exhibit enhanced angiogenic and invasive properties. (B) Immunohistochemical staining using an antibody directed against the mitosis and proliferation marker Ki-67 showing less frequent and weaker mitotic activity in SW480-gRNA-B-all-T compared to SW480-T cells (black scale bars in the bottom right corners, 20 µm).

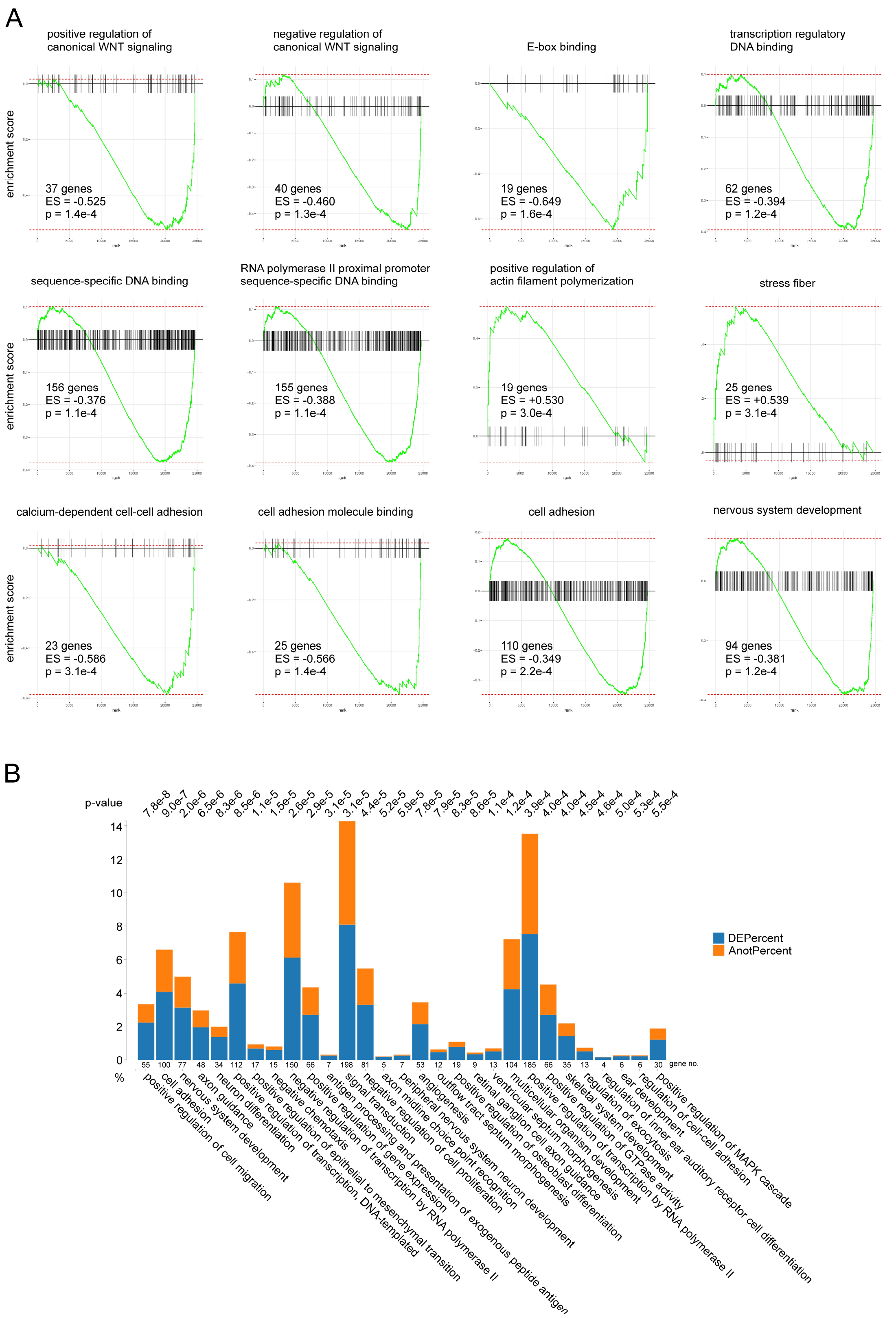
A
positive regulation of canonical WNT signaling
37 genes
ES = -0.525
p = 1.4e-4
negative regulation of canonical WNT signaling
40 genes
ES = -0.460
p = 1.3e-4
E-box binding
19 genes
ES = -0.649
p = 1.6e-4
transcription regulatory DNA binding
62 genes
ES = -0.394
p = 1.2e-4
sequence-specific DNA binding
156 genes
ES = -0.376
p = 1.1e-4
RNA polymerase II proximal promoter sequence-specific DNA binding
155 genes
ES = -0.388
p = 1.1e-4
positive regulation of actin filament polymerization
19 genes
ES = +0.530
p = 3.0e-4
stress fiber
25 genes
ES = +0.539
p = 3.1e-4
calcium-dependent cell-cell adhesion
23 genes
ES = -0.586
p = 3.1e-4
cell adhesion molecule binding
25 genes
ES = -0.566
p = 1.4e-4
cell adhesion
110 genes
ES = -0.349
p = 2.2e-4
nervous system development
94 genes
ES = -0.381
p = 1.2e-4
enrichment score
B
p-value
7.8e-8
9.0e-7
2.0e-6
6.5e-6
8.3e-6
8.5e-6
1.1e-5
1.5e-5
2.6e-5
2.9e-5
3.1e-5
3.1e-5
4.4e-5
5.2e-5
5.9e-5
7.8e-5
7.9e-5
8.3e-5
8.6e-5
1.1e-4
1.2e-4
3.9e-4
4.0e-4
4.0e-4
4.5e-4
4.6e-4
5.0e-4
5.3e-4
5.5e-4
%
14
12
10
8
6
4
2
0
55
100
77
48
34
112
17
15
150
66
7
198
81
5
7
53
12
19
9
13
104
185
66
35
13
4
6
6
30
gene no.
DEPercent
AnotPercent
positive regulation of cell migration
cell adhesion
nervous system development
axon guidance
neuron differentiation
positive regulation of transcription, DNA-templated
negative chemotaxis
positive regulation of epithelial to mesenchymal transition
negative regulation of transcription by RNA polymerase II
positive regulation of gene expression
antigen processing and presentation of exogenous peptide antigen
signal transduction
negative regulation of cell proliferation
axon midline choice point recognition
peripheral nervous system neuron development
angiogenesis
outflow tract septum morphogenesis
positive regulation of osteoblast differentiation
retinal ganglion cell axon guidance
ventricular septum morphogenesis
multicellular organism development
positive regulation of transcription by RNA polymerase II
positive regulation of GTPase activity
skeletal system development
regulation of exocytosis
ear development
regulation of inner ear auditory receptor cell differentiation
regulation of cell-cell adhesion
positive regulation of MAPK cascade

Figure S6. (A) Representative gene set enrichment analyses (GSEA) from SW480 versus SW480-gRNA-B-all expressed mRNAs, encoding proteins implicated in pathways such as regulation of WNT signaling, sequence-specific DNA binding, transcriptional regulation, regulation of actin filament polymerization and stress fibers, cell adhesion, and nervous system development. A positive enrichment score (ES) indicates that the majority of each set is upregulated in BASP1-expressing cells, whereas a negative value indicates preponderant downregulation. The degrees of enrichment, relevant p-values, and gene numbers of each set are indicated. (B) Gene ontology (GO) analysis of SW480 and SW480-gRNA-B-all cells ranked according to their p-values. The differential expression (DEPercent) degrees in a set of annotated genes (AnotPercent) from a distinct cluster are indicated by stacked blue and orange bars, respectively. High proportion of differential gene expression is observed for pathways like cell migration, cell adhesion, neuronal development, transcriptional regulation, or MAPK signaling.

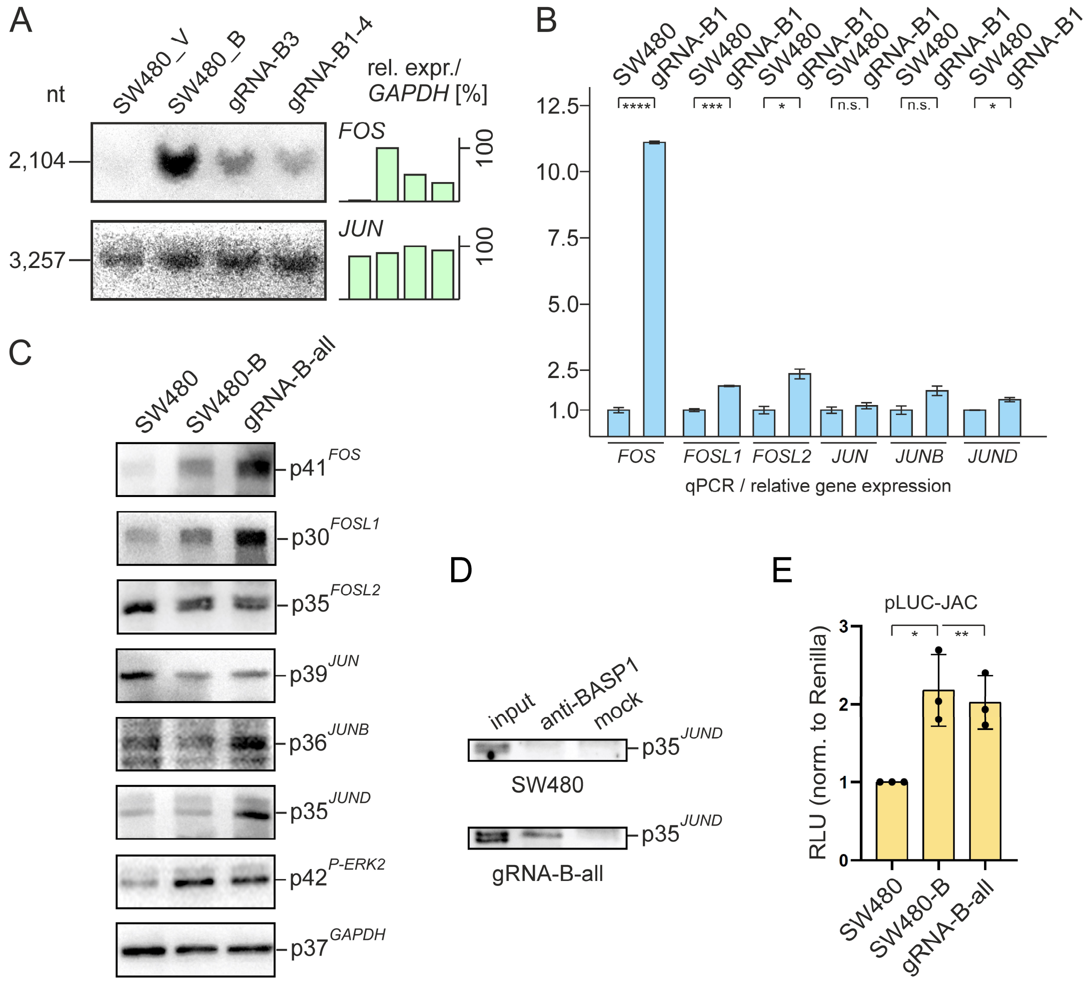

Figure S7. Activation of AP-1 in BASP1-expressing SW480 cells. (A) Northern analysis using the RNA samples applied in Figure 2C and DNA probes specific for the human *FOS* and *JUN* mRNAs. Normalization was done on *GAPDH* expression (cf. Figure 2C). (B) Quantitative PCR (qPCR) to test for expression of genes encoding AP-1 components from the JUN and FOS protein families (*P < 0.05, ***P < 0.001, ****P < 0.0001, n.s. not significant). (C) Immunoblot analysis to monitor expression of JUN and FOS protein family members and of phosphorylated extracellular signal-regulated MAP kinase 1 (P-ERK2). (D) Co-immunoprecipitation using extracts from SW480 and SW480-gRNA-B-all cells and anti-BASP1 as first antibody to test for physical interaction between BASP1 and JUND. (E) Transcriptional activation of an AP-1 reporter plasmid (pLUC-JAC) containing two AP-1 binding sites (17) in SW480 and BASP1-expressing SW480 (B, gRNA-B-all) cells (*P < 0.05, **P < 0.01).

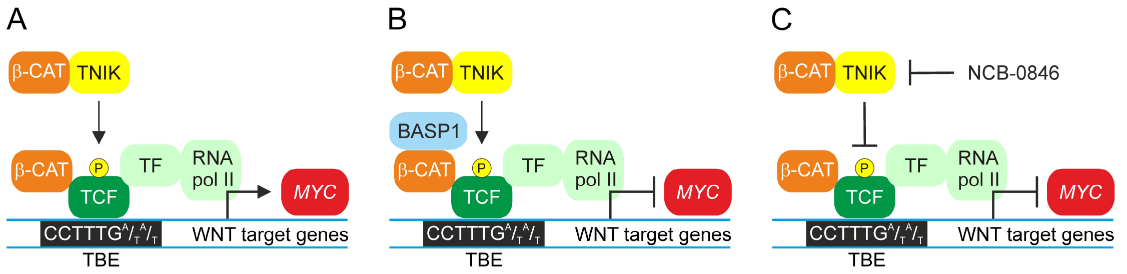

Figure S8. Schematic depiction how BASP1 and the chemical inhibitor NCB-0846 may interfere with transcriptional *MYC* activation in colorectal cancer cells. (A) In activated WNT signaling TNIK interacts with β-catenin (β-CAT) leading to the formation of a complex, which translocates into the nucleus where TNIK phosphorylates TCF7L2. This event leads to transcriptional activation of WNT target genes such as *MYC* (20). (B) Overexpressed BASP1 interacts with β-catenin thereby leading to transcriptional *MYC* repression. TNIK-mediated phosphorylation may still occur although this kinase is downregulated in the presence of BASP1 (cf. Figure 4). (C) TNIK inhibition by NCB-0846 prevents TCF7L2 phosphorylation thereby causing suppression of *MYC* transcription.
